# Age-related changes of color visual acuity in normal eyes

**DOI:** 10.1371/journal.pone.0260525

**Published:** 2021-11-29

**Authors:** Sho Yokoyama, Yoshiki Tanaka, Takashi Kojima, Rie Horai, Yukihito Kato, Hideki Nakamura, Hiroyuki Sato, Mari Mitamura, Kiyoshi Tanaka, Kazuo Ichikawa

**Affiliations:** 1 Department of Ophthalmology, Japan Community Healthcare Organization Chukyo Hospital, Nagoya, Japan; 2 Chukyo Eye Clinic, Nagoya, Japan; 3 Department of Ophthalmology, Keio University School of Medicine, Tokyo, Japan; 4 Meito Eye Clinic, Nagoya, Japan; 5 Satoh Yuya Eye Clinic, Sendai, Japan; 6 Faculty of Engineering, Shinshu University, Nagano, Japan; University of Oxford, UNITED KINGDOM

## Abstract

**Purpose:**

To evaluate the age-related change in color visual acuity (CVA) in normal eyes.

**Methods:**

In total, 162 normal eyes (162 subjects, women: 52, men: 110, age range: 15–68 years) with best-corrected visual acuity (BCVA) ≥20/13 were enrolled. Fifteen colors from the New Color Test (chroma 6) were applied to Landolt “*C*” rings, white point D65 was applied as background, and a luminance of 30 cd/m^2^ was set for both the rings and the background. These rings were used to measure the chromatic spatial discrimination acuity as the CVA value, while changing the stimulus size. Correlations of the CVA value of each color and age were evaluated. Mean CVA values of all 15 colors (logarithm of the minimum angle of resolution) were compared between age groups in 10-year increments.

**Results:**

Nine CVA values (red, yellow-red, red-yellow, green, blue-green, green-blue, purple, red-purple, and purple-red) were negatively correlated with age (all *p*<0.05); the remaining six (yellow, green-yellow, yellow-green, blue, purple-blue, and blue-purple), as well as BCVA were not. The age groups with the best to worst mean CVA values of 15 colors were as follows: 20–29 (mean ± standard deviation, 0.303 ± 0.113), 30–39 (0.324 ± 0.096), 10–19 (0.333 ± 0.022), 50–59 (0.335 ± 0.078), 40–49 (0.339 ± 0.096), and 60–69 (0.379 ± 0.125) years. There were statistically significant differences between mean CVA values of the following groups: 20–29 and 40–49 years; 20–29 and 60–69 years; 30–39 and 60–69 years (all *p*<0.01).

**Conclusions:**

The CVA values related to the medium/long-wavelength-sensitive cones were more susceptible to aging than those related to the short-wavelength-sensitive cones. This differed from previous reports, and may be related to the difference in the range of foveal cone function evaluated with each examination.

## Introduction

Human visual perception consists of spatial characteristics, temporal characteristics, and color vision. Visual acuity (VA) is defined as spatial discrimination ability and is represented by the minimum visual angle at which two separate objects can be discriminated [1]. VA test is the most common way to measure visual function. The target stimulus of a VA test is usually composed of an achromatic color with a luminance contrast of 100% (luminance 100%, white; luminance 0%, black). In other words, the black-and-white VA test is used to evaluate the achromatic spatial discrimination at 100% luminance contrast; hence, only a small portion of the broad range of visual function is evaluated with a VA test. However, the world is not achromatic, and a VA test that is more in line with real world is required, incorporating color information. In addition, as color vision deficiency develops from the early stages of various diseases, mainly in retinal and optic nerve disease [2–5], color vision tests have been useful adjuncts in the diagnosis and management of many ocular diseases [6]. By incorporating color information into the conventional VA test, it may be possible to evaluate visual function in a way that is more relevant to daily life, and to detect diseases earlier than with the conventional black-and-white VA tests. Therefore, we developed a novel visual function testing system that can be used to display the target stimulus and background in any color on a liquid crystal display (LCD) monitor with high color reproducibility [7, 8]. Our research group has adopted the Landolt “*C*” ring as the target stimulus; its direction and presentation size can be changed by using a personal computer (PC). In our previous study using this testing system, 15 colored Landolt rings and an achromatic background were used, and the VA of each color was measured in normal eyes of individuals in their 20s while changing the size of the colored Landolt rings [9]. In that study, there were no differences in VA among the different colors when the luminance values of the Landolt rings and background were different; however, statistically significant differences became apparent when the luminance values of the Landolt rings and background were equalized. That is, when the luminance value of the Landolt rings and background is equalized, the chromatic spatial discrimination acuity at a constant saturation and luminance can be evaluated. We defined color visual acuity (CVA) as “chromatic spatial discrimination acuity at a constant saturation and luminance,” as opposed to the “achromatic spatial discrimination acuity at 100% luminance contrast” that is measured with conventional black-and-white VA tests.

It is known that color discrimination gradually deteriorates with age. It has been reported that both the medium/long-wavelength-sensitive cones (M/L-cones), which relates to red-green (RG)-axis colors, and the short-wavelength-sensitive cones (S-cones), which relates to blue-yellow (BY)-axis colors, can be affected [6], and that the S-cones are more susceptible to the effects of aging than the M/L-cones are [10, 11].

We hypothesized that CVA changes with age even in normal eyes, and that the degree of such change differs among colors. To test this hypothesis, we measured CVA values for 15 colors in normal eyes and evaluated the changes in each CVA due to aging as well as the differences in CVA values in each age group.

## Materials and methods

This was an observational, prospective study, including normal eyes for which CVA tests were performed from January 2010 to December 2017 at Japan Community Healthcare Organization Chukyo Hospital, Chukyo Eye Clinic, or Satoh Yuya Eye Clinic. This research was approved by the Institutional Review Boards of Chukyo Medical and Japan Community Healthcare Organization Chukyo Hospital, and it adhered to the tenets of the Declaration of Helsinki.

Participants were recruited from volunteers and patients with normal eye(s) who visited the clinic for an eye-screening test or were followed-up for disease of the other eye. We obtained written informed consent from each participant after explanation of the nature of the study. The inclusion criteria were as follows: age 10 to 69; best-corrected visual acuity (BCVA) better than or equal to 20/13; no crystalline lens sclerosis, cortical opacity, or posterior subcapsular opacity within the natural pupil diameter when examined under a slit-lamp microscope; and no apparent optic nerve/macular abnormalities when examined using ophthalmic fundoscopy. Participants with congenital color vision deficiency using Standard Pseudoisochromatic Plates Part-3 (SPP-3; Igaku-Shoin Ltd., Tokyo, Japan) [12, 13] were excluded. Participants who missed at least one target figure in plate nos. 2–4 of SPP-3 (for detecting RG-axis color blindness) were additionally examined using Nagel’s anomaloscope (Nagel Type I; Schmidt and Haensch GmbH and Co., Berlin, Germany) to rule out congenital color vision deficiency. Participants who did not match near [40, 15] scales in the RG mixture field and yellow reference field, respectively, were excluded from this study as they could have had congenital color vision deficiency. CVA data was used from participants’ normal eye; where both eyes were normal, data from the right eye were used. Participants were grouped in 10-year age increments, from 10 to 69 years, for comparisons between age groups.

In a preliminary study of 50 subjects, we calculated the required number of samples from the correlation coefficient (*r*) between the CVA values of red and age using G*Power software (Heinrich Heine University of Duesseldorf, Germany) [14]. The required sample size was calculated as 160 when the detection power was set to 0.95.

### Color visual acuity testing system and measurement

**Fig 1:**
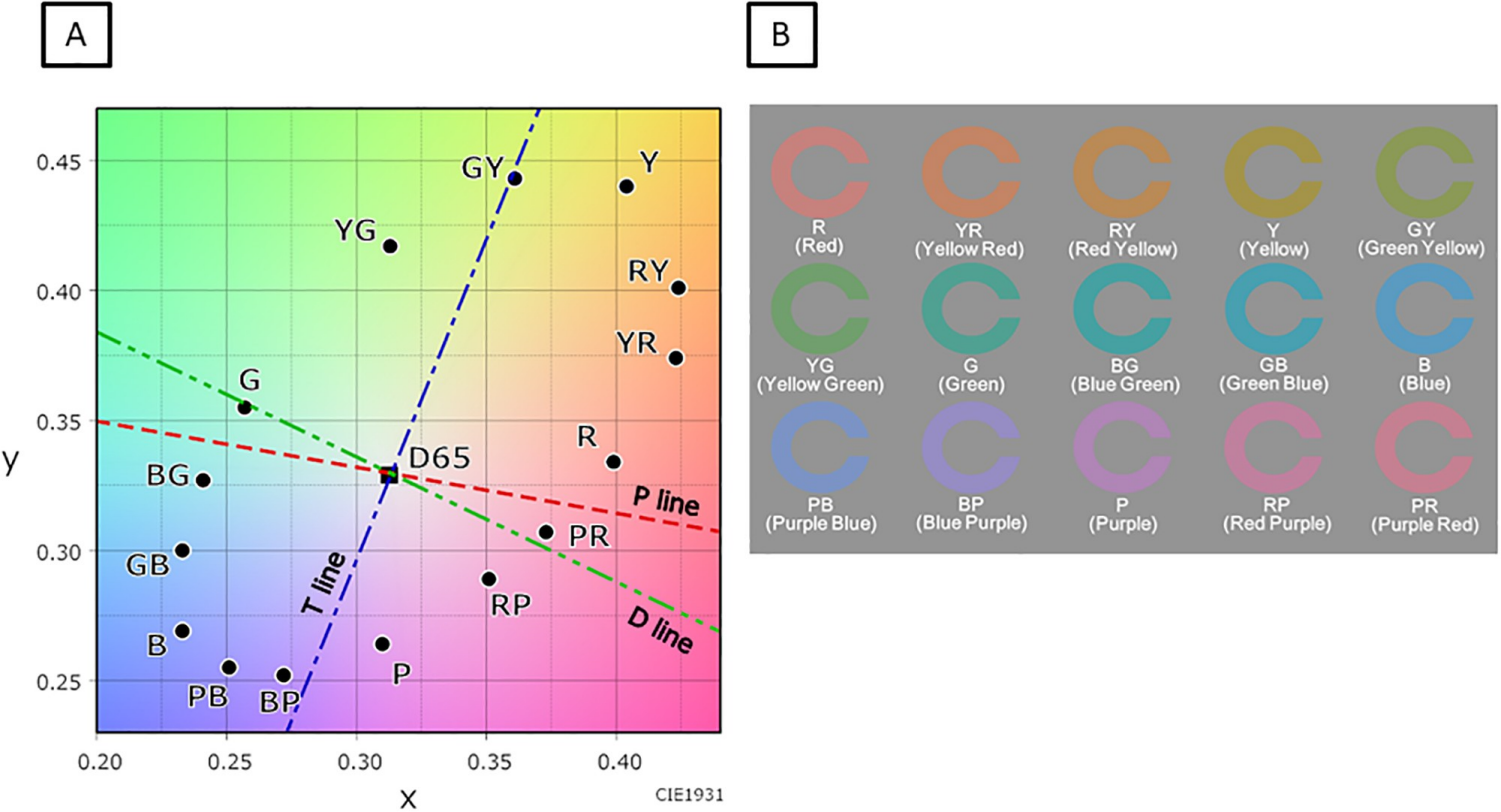
The chromaticity points of Landolt rings of 15 different colors used as target stimuli in this study. A: The chromaticity points of 15 colors of the New Color Test at chroma 6 and the white point D65 on the Commission Internationale de l’Eclairage (CIE) 1931 chromaticity diagram, with the three confusion lines: deuteranopic (D), protanopic (P), and tritanopic (T) lines. B: Landolt rings of 15 different colors (according to the New Color Test, at chroma 6). Both the Landolt rings and background have a luminance of 30 cd/m^2^.

The detail of the CVA system and examination methods were described in our previous study [9]. Briefly, the CVA testing system consists of a PC, on which to run the management software, and a ColorEdge CG245W LCD monitor (EIZO Corp., Ishikawa, Japan), as it can achieve color calibration with high precision. The Landolt “*C*” ring was used as the target stimulus and the gap size was set to 1/5 of the overall Landolt ring size. The 15 colors of chroma 6 in the New Color Test (Luneau Ophthalmology, Paris, France), a color arrangement test that includes various saturations of chromas 2, 4, 6, and 8 [15], were used for the Landolt rings, and white point D65 was used for the background color (Fig 1A). The luminance values of both the stimulus and the background were set to 30 cd/m^2^. Fig 1B contains the 15 colored Landolt rings: red (R), yellow-red (YR), red-yellow (RY), yellow (Y), green-yellow (GY), yellow-green (YG), green (G), blue-green (BG), green-blue (GB), blue (B), purple-blue (PB), blue-purple (BP), purple (P), red-purple (RP), and purple-red (PR). The Landolt ring sizes were pre-set for measuring a range of decimal VA from 0.04 to 1.5 (logarithm of the minimum angle of resolution [logMAR] VA, 1.40 to -0.18). The maximum presentation time of each stimulus was set to 5 s, with an interval time of 2 s during which only the background color was displayed. Prior to the start of each test, the monitor was calibrated. Before starting the examination, participants’ eyes were allowed to adapt to the background color on the screen for 5 min. The participants were seated at a distance of 3 m from the LCD test screen in a dark room and the refractive error was fully corrected by using glasses. For all participants, each CVA was measured separately for each eye, and the right eye was always tested first. The CVA test was performed with the same up-and-down method used in conventional VA measurements. The Landolt ring was presented to the subject with its gap in one of four directions: up, down, right, or left. If the subject answered correctly, the stimulus size was reduced by one step. If the answer was incorrect or not supplied, the stimulus size was increased by one step. The CVA value was calculated from the minimum Landolt ring size for which the subjects answered correctly more than two times out of a maximum of five times. In S1 Video, the CVA testing system is demonstrated by using an R and a BP Landolt ring.

### Statistical analysis

The BCVA and CVA values were converted to logMAR values for statistical analysis. Spearman’s correlation coefficient test was applied to evaluate the correlation of BCVA and each of the 15 CVA values with age. Dunn’s post-hoc test was used for comparison of the CVA values between the 15 colors and an F-test was used for comparison of the variances of the CVA values between the 15 colors. Kruskal-Wallis and Mann-Whitney U tests were used to determine differences in CVA values between the age groups. Statistical analyses were performed by using the R statistical language (R Development Core Team. R: A language and environment for statistical computing. R Foundation for Statistical Computing, Vienna, Austria) and GraphPad Prism version 9.0 for Windows (GraphPad Software, San Diego, CA, USA). A *p*-value less than 5% was considered statistically significant.

## Results

### Baseline characteristics

**Fig 2:**
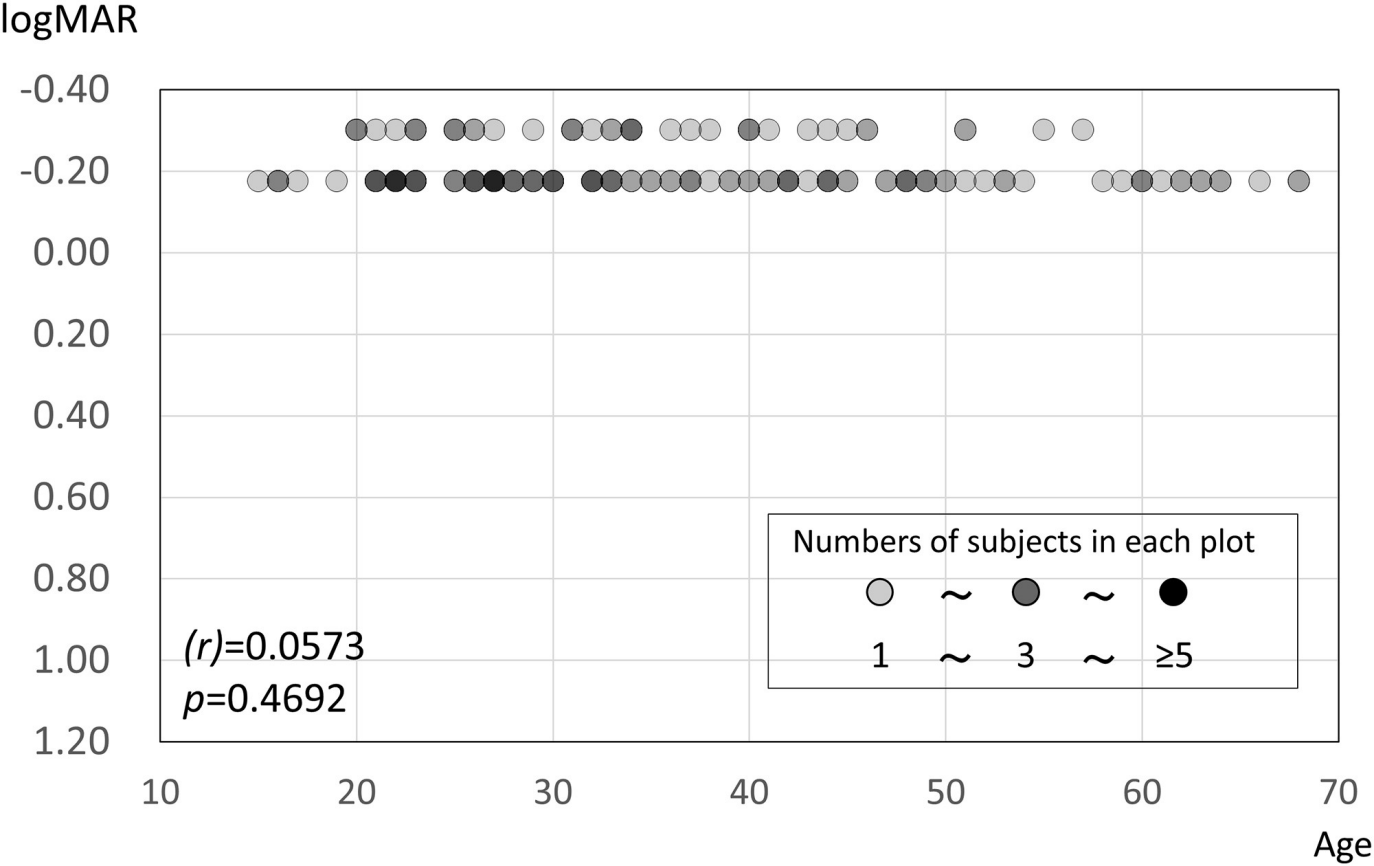
Relationship between best-corrected visual acuity (BCVA) and age for all 162 participants. BCVA was not correlated with age (Spearman’s correlation coefficient, *r* [95% confidence interval] = 0.0573 [-0.1204 to 0.2140], *p* = 0.4692).

One hundred sixty-two participants (one eye each; women: 52; men: 110), with a mean age of 35.9 ± 12.9 years (range: 15–68 years), were enrolled in this study (Table 1). The BCVA was 20/13 in 121 eyes and 20/10 in 41 eyes, and the mean BCVA in all 162 eyes was 0.105 ± 0.324 (logMAR, mean ± standard deviation [SD]). There was no correlation between BCVA and age (Spearman’s correlation coefficient, *r =* 0.0573, *p* = 0.4692) (Fig 2).

**Table 1:** Participants’ demographic information in each age group.

Age	Numbers	Male	Female	Age (mean ± SD, median)
**10–19**	6	6	0	16.5 ± 1.4, 16
**20–29**	58	32	26	24.6 ± 2.8, 25
**30–39**	39	27	12	33.8 ± 2.7, 33
**40–49**	33	27	6	44.2 ± 3.0, 44
**50–59**	13	9	4	53.4 ± 3.0, 53
**60–69**	13	9	4	63.2 ± 2.8, 63
**Total**	162	110	52	35.9 ± 12.9, 33

SD: standard deviation.

### Correlation between CVA values and age

**Fig 3:**
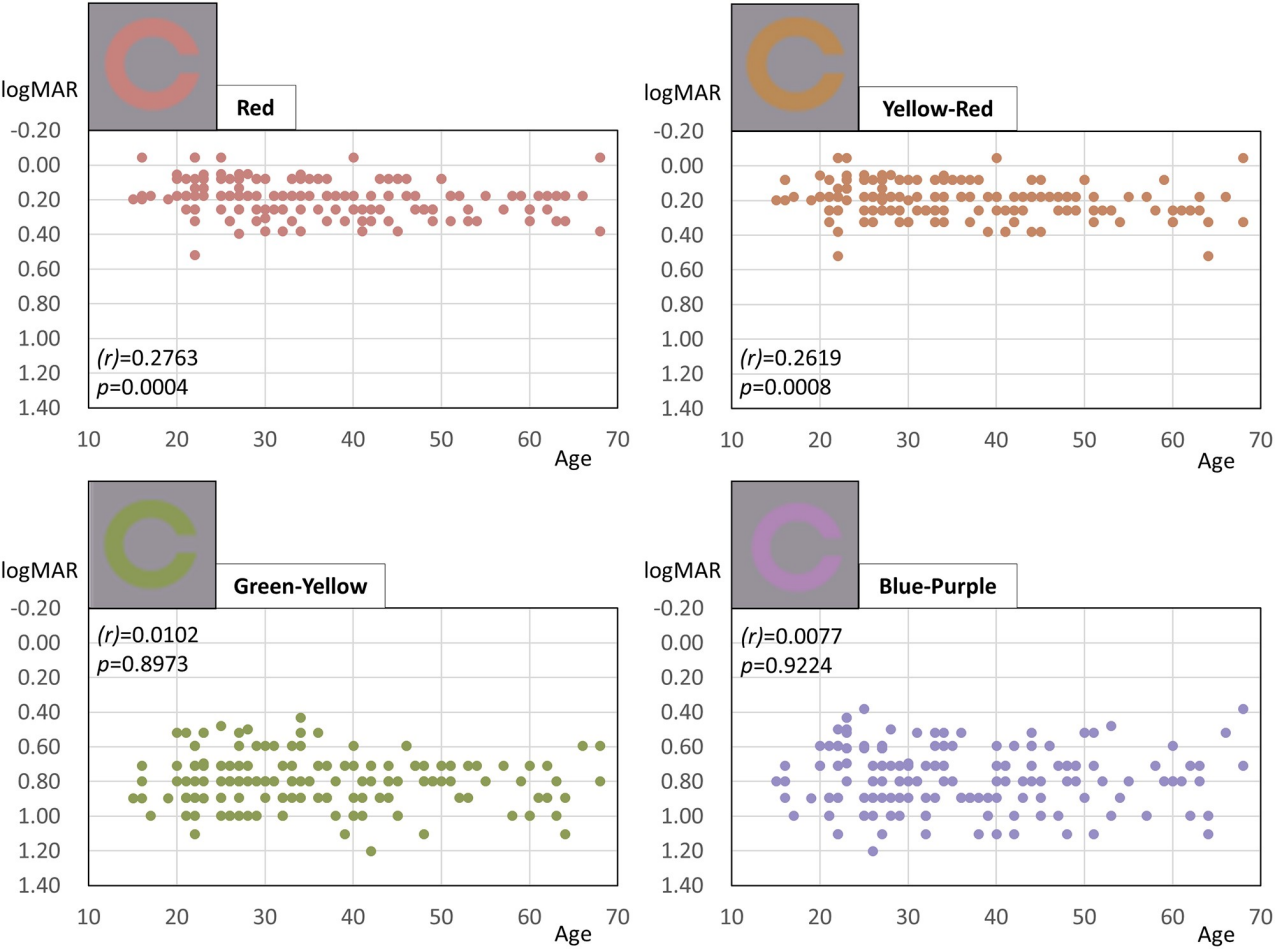
Four representative scatterplots of color visual acuity (CVA), those of red (R), yellow-red (YR), green-yellow (GY), and blue-purple (BP), versus age, for all 162 participants. The R- and YR-CVA values were negatively correlated with age (Spearman’s correlation coefficient, *r* = 0.2763, *p =* 0.0004 and *r* = 0.2619, *p =* 0.0008, respectively), whereas GY- and BP-CVA were not correlated with age (*r* = 0.0102, *p =* 0.8973 and *r* = 0.0077, *p* = 0.9224, respectively).

Table 2 shows the correlation coefficients (*r* [95% confidence interval]) and *p*-values of Spearman’s correlation test between each CVA and age. Among the 15 CVA values, 9 were negatively correlated with age. R-CVA had the strongest negative correlation with age (*r* = 0.2763, *p* = 0.0004). The negative correlation between CVA and age, from strongest to weakest, were as follows: R-, YR-, RY-, PR-, GB-, P-, RP-, G-, and BG-CVA. The remaining six CVA values (Y, GY, YG, B, PB, and BP) were not correlated with age. Fig 3 contains four representative scatterplots of CVA values versus age for all 162 participants: the two CVA values with the strongest negative correlation with age (R-CVA and YR-CVA), and two of the six that were not correlated with age (GY-CVA and BP-CVA). The other scatterplots of each CVA value versus age for all 162 participants are contained in S1–S11 Figs (RY-, Y-, GY-, YG-, G-, BG-, GB-, B-, P-, RP-, and PR-CVA).

**Table 2:** Results of correlation analysis between each color visual acuity and age.

Color	Correlation coefficient, *r* (95% CI)	*p*-value
**Red (R)**	0.2763 (0.1230 to 0.4168)	0.0004
**Yellow-Red (YR)**	0.2619 (0.1077 to 0.4038)	0.0008
**Red-Yellow (RY)**	0.2476 (0.0925 to 0.3909)	0.0015
**Yellow (Y)**	0.0940 (-0.0657 to 0.2490)	0.2341
**Green-Yellow (GY)**	0.0102 (-0.1487 to 0.1687)	0.8973
**Yellow-Green (YG)**	0.1255 (-0.0339 to 0.2787)	0.1116
**Green (G)**	0.1884 (0.0306 to 0.3370)	0.0164
**Blue-Green (BG)**	0.1641 (0.0055 to 0.3146)	0.0369
**Green-Blue (GB)**	0.2193 (0.0628 to 0.3653)	0.0051
**Blue (B)**	0.0984 (-0.0613 to 0.2531)	0.2130
**Purple-Blue (PB)**	0.0632 (-0.0965 to 0.2197)	0.4244
**Blue-Purple (BP)**	0.0077 (-0.1512 to 0.1662)	0.9224
**Purple (P)**	0.2117 (0.0548 to 0.3584)	0.0068
**Red-Purple (RP)**	0.2055 (0.0484 to 0.3527)	0.0087
**Purple-Red (PR)**	0.2432 (0.0879 to 0.3870)	0.0018

Correlation coefficients and *p*-values were analyzed by using Spearman’s correlation test.

CI: Confidence interval.

### Comparison of CVA values between 15 colors

**Fig 4:**
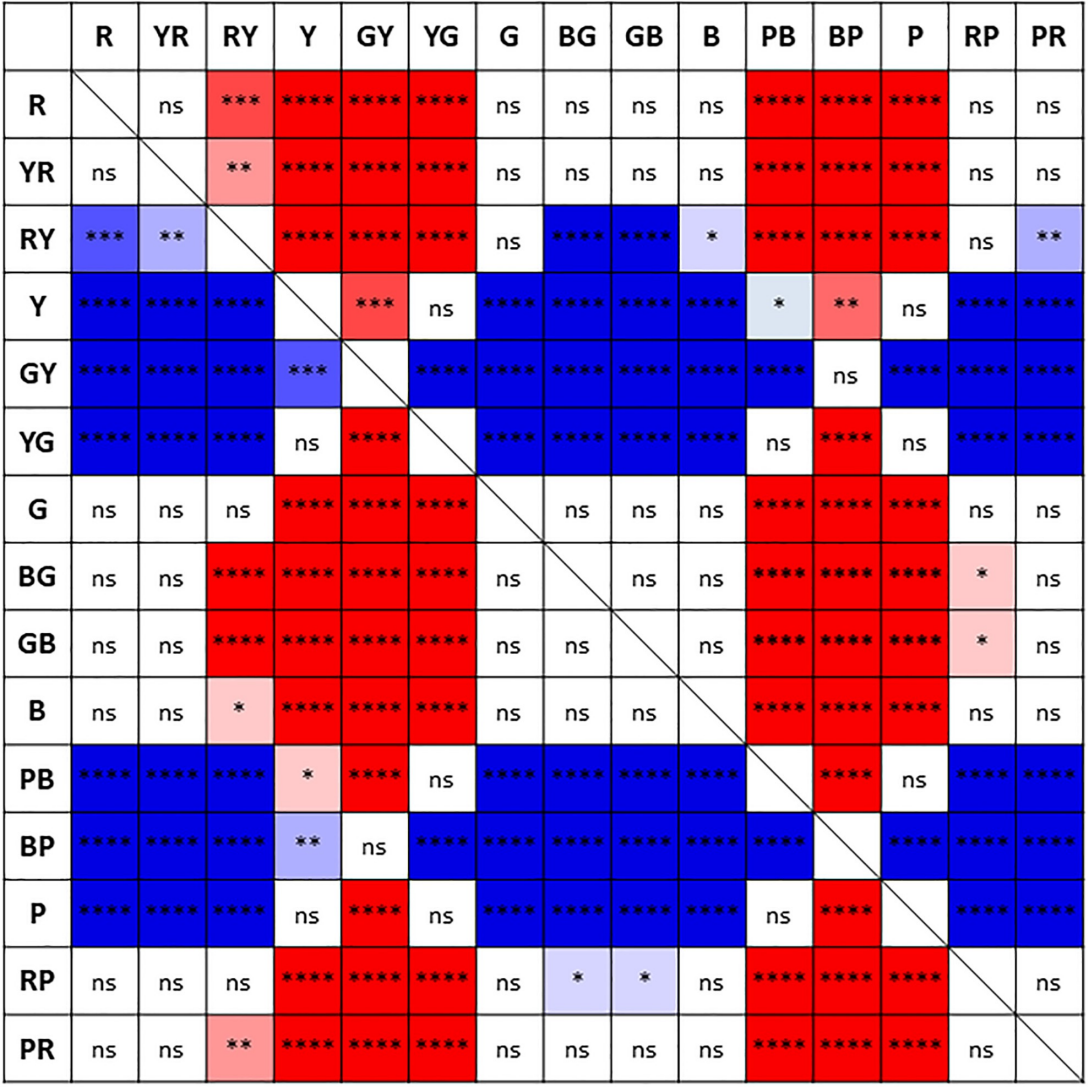
Comparison of the color visual acuity (CVA) values between the 15 colors. The *p*-values of Dunn’s post-hoc test for comparison between CVA values for the 15 colors in all participants are summarized (ns: no significant difference, *p<0.05, **p<0.01, ***p<0.001, ****p<0.0001). The cells are shown in warmer colors (red shades) when the colors arranged vertically have significantly higher CVA values than the colors arranged horizontally, and the cells are shown in cooler colors (blue shades) when those values are significantly lower. R: red, YR: yellow-red, RY: red-yellow, Y: yellow, GY: green-yellow, YG: yellow-green, G: green, BG: blue-green, GB: green-blue, B: blue, PB: purple-blue, BP: blue-purple, P: purple, RP: red-purple, PR: purple-red.

Table 3 summarizes the CVA values for the 15 colors (logMAR, mean ± SD, median [interquartile range]) for each age group and all participants. The best to worst mean CVA values in all participants were as follows: BG-CVA (mean ± SD, 0.180 ± 0.116), GB-CVA (0.182 ± 0.111), R-CVA (0.188 ± 0.101), YR-CVA (0.193 ± 0.102), PR-CVA (0.200 ± 0.113), B-CVA (0.200 ± 0.116), G-CVA (0.209 ± 0.119), RP-CVA (0.222 ± 0.120), RY-CVA (0.240 ± 0.112), PB-CVA (0.340 ± 0.128), YG-CVA (0.346 ± 0.130), P-CVA (0.368 ± 0.122), Y-CVA (0.432 ± 0.134), BP-CVA (0.777 ± 0.177), and GY-CVA (0.805 ± 0.146). Fig 4 summarizes the comparison of CVA values between the 15 colors in all participants. The highest mean CVA value was obtained for BG, which was significantly higher than for RY, Y, GY, YG, PB, BP, P, and RP. The lowest mean CVA value was obtained for GY, which was significantly lower than for all other colors except for BP.

**Table 3:** Results of the 15 color visual acuities in each age group.

Color		Age						
		10–19	20–29	30–39	40–49	50–59	60–69	All
**Red (R)**	Mean ± SD	0.15 ± 0.10	0.16 ± 0.10	0.20 ± 0.09	0.22 ± 0.10	0.21 ± 0.08	0.23 ± 0.11	0.188 ± 0.101
	Median (IQR)	0.19 (0.12 to 0.20)	0.18 (0.08 to 0.18)	0.18 (0.08 to 0.25)	0.25 (0.18 to 0.25)	0.18 (0.18 to 0.29)	0.25 (0.18 to 0.32)	0.18 (0.12 to 0.25)
**Yellow-Red (YR)**	Mean ± SD	0.15 ± 0.06	0.17 ± 0.11	0.19 ± 0.09	0.22 ± 0.09	0.21 ± 0.09	0.26 ± 0.13	0.193 ± 0.102
	Median (IQR)	0.19 (0.08 to 0.20)	0.18 (0.08 to 0.25)	0.18 (0.08 to 0.25)	0.18 (0.18 to 0.25)	0.25 (0.13 to 0.25)	0.25 (0.22 to 0.32)	0.18 (0.12 to 0.25)
**Red-Yellow (RY)**	Mean ± SD	0.19 ± 0.08	0.21 ± 0.11	0.24 ± 0.11	0.27 ± 0.12	0.26 ± 0.09	0.28 ± 0.14	0.240 ± 0.112
	Median (IQR)	0.19 (0.12 to 0.27)	0.20 (0.13 to 0.27)	0.25 (0.18 to 0.32)	0.25 (0.18 to 0.32)	0.25 (0.22 to 0.32)	0.25 (0.18 to 0.35)	0.25 (0.18 to 0.32)
**Yellow (Y)**	Mean ± SD	0.49 ± 0.13	0.40 ± 0.14	0.44 ± 0.11	0.45 ± 0.13	0.44 ± 0.12	0.45 ± 0.16	0.432 ± 0.134
	Median (IQR)	0.51 (0.39 to 0.61)	0.40 (0.31 to 0.48)	0.43 (0.32 to 0.52)	0.43 (0.38 to 0.52)	0.43 (0.35 to 0.56)	0.43 (0.35 to 0.52)	0.43 (0.32 to 0.52)
**Green-Yellow (GY)**	Mean ± SD	0.87 ± 0.10	0.79 ± 0.16	0.79 ± 0.15	0.83 ± 0.14	0.79 ± 0.09	0.84 ± 0.16	0.805 ± 0.146
	Median (IQR)	0.90 (0.78 to 0.92)	0.80 (0.71 to 0.90)	0.80 (0.71 to 0.89)	0.80 (0.71 to 0.89)	0.80 (0.71 to 0.85)	0.90 (0.71 to 1.00)	0.80 (0.71 to 0.89)
**Yellow-Green (YG)**	Mean ± SD	0.41 ± 0.13	0.33 ± 0.14	0.32 ± 0.13	0.35 ± 0.11	0.33 ± 0.08	0.45 ± 0.15	0.346 ± 0.130
	Median (IQR)	0.39 (0.31 to 0.53)	0.32 (0.25 to 0.38)	0.32 (0.25 to 0.43)	0.32 (0.25 to 0.43)	0.32 (0.29 to 0.41)	0.38 (0.38 to 0.56)	0.32 (0.25 to 0.43)
**Green (G)**	Mean ± SD	0.24 ± 0.09	0.18 ± 0.13	0.21 ± 0.11	0.21 ± 0.10	0.22 ± 0.10	0.30 ± 0.12	0.209 ± 0.119
	Median (IQR)	0.28 (0.15 to 0.31)	0.18 (0.08 to 0.25)	0.18 (0.08 to 0.25)	0.18 (0.18 to 0.25)	0.25 (0.13 to 0.32)	0.32 (0.25 to 0.32)	0.18 (0.13 to 0.31)
**Blue-Green (BG)**	Mean ± SD	0.22 ± 0.09	0.15 ± 0.12	0.18 ± 0.11	0.18 ± 0.11	0.19 ± 0.11	0.25 ± 0.12	0.180 ± 0.116
	Median (IQR)	0.23 (0.15 to 0.31)	0.18 (0.08 to 0.25)	0.18 (0.08 to 0.25)	0.18 (0.08 to 0.25)	0.18 (0.13 to 0.25)	0.25 (0.18 to 0.35)	0.18 (0.08 to 0.25)
**Green-Blue (GB)**	Mean ± SD	0.16 ± 0.11	0.16 ± 0.11	0.17 ± 0.12	0.19 ± 0.10	0.20 ± 0.07	0.27 ± 0.14	0.182 ± 0.111
	Median (IQR)	0.20 (0.12 to 0.21)	0.18 (0.08 to 0.21)	0.18 (0.08 to 0.25)	0.18 (0.18 to 0.25)	0.18 (0.18 to 0.25)	0.25 (0.22 to 0.35)	0.18 (0.08 to 0.25)
**Blue (B)**	Mean ± SD	0.20 ± 0.08	0.19 ± 0.12	0.19 ± 0.11	0.20 ± 0.11	0.19 ± 0.08	0.29 ± 0.15	0.200 ± 0.116
	Median (IQR)	0.20 (0.15 to 0.27)	0.18 (0.12 to 0.25)	0.18 (0.08 to 0.25)	0.18 (0.18 to 0.25)	0.18 (0.13 to 0.25)	0.32 (0.18 to 0.38)	0.18 (0.16 to 0.25)
**Purple-Blue (PB)**	Mean ± SD	0.39 ± 0.08	0.33 ± 0.15	0.34 ± 0.11	0.34 ± 0.12	0.32 ± 0.09	0.40 ± 0.15	0.340 ± 0.128
	Median (IQR)	0.39 (0.32 to 0.42)	0.32 (0.24 to 0.38)	0.32 (0.25 to 0.38)	0.32 (0.25 to 0.38)	0.32 (0.25 to 0.41)	0.38 (0.32 to 0.50)	0.32 (0.25 to 0.39)
**Blue-Purple (BP)**	Mean ± SD	0.85 ± 0.10	0.76 ± 0.19	0.79 ± 0.16	0.78 ± 0.16	0.79 ± 0.20	0.76 ± 0.20	0.777 ± 0.177
	Median (IQR)	0.85 (0.78 to 0.92)	0.71 (0.60 to 0.89)	0.80 (0.71 to 0.89)	0.71 (0.65 to 0.89)	0.80 (0.61 to 0.94)	0.80 (0.65 to 0.90)	0.80 (0.61 to 0.89)
**Purple (P)**	Mean ± SD	0.34 ± 0.06	0.34 ± 0.14	0.37 ± 0.10	0.40 ± 0.13	0.39 ± 0.09	0.41 ± 0.12	0.368 ± 0.122
	Median (IQR)	0.35 (0.29 to 0.39)	0.32 (0.25 to 0.43)	0.32 (0.32 to 0.43)	0.38 (0.32 to 0.52)	0.38 (0.18 to 0.43)	0.38 (0.35 to 0.52)	0.38 (0.25 to 0.43)
**Red-Purple (RP)**	Mean ± SD	0.20 ± 0.06	0.20 ± 0.14	0.22 ± 0.10	0.25 ± 0.10	0.27 ± 0.11	0.25 ± 0.13	0.222 ± 0.120
	Median (IQR)	0.20 (0.17 to 0.25)	0.18 (0.08 to 0.32)	0.18 (0.18 to 0.31)	0.25 (0.18 to 0.32)	0.32 (0.22 to 0.32)	0.25 (0.18 to 0.32)	0.25 (0.18 to 0.32)
**Purple-Red (PR)**	Mean ± SD	0.14 ± 0.10	0.17 ± 0.12	0.21 ± 0.11	0.20 ± 0.11	0.24 ± 0.10	0.24 ± 0.12	0.200 ± 0.113
	Median (IQR)	0.15 (0.09 to 0.21)	0.18 (0.08 to 0.25)	0.18 (0.08 to 0.31)	0.18 (0.13 to 0.32)	0.25 (0.18 to 0.29)	0.25 (0.18 to 0.32)	0.18 (0.08 to 0.25)
**All colors**	Mean ± SD	0.333 ± 0.022	0.303 ± 0.113	0.324 ± 0.096	0.339 ± 0.096	0.335 ± 0.078	0.379 ± 0.125	0.324 ± 0.105
	Median (IQR)	0.25 (0.20 to 0.39)	0.25 (0.13 to 0.38)	0.25 (0.18 to 0.38)	0.25 (0.18 to 0.43)	0.25 (0.18 to 0.38)	0.32 (0.25 to 0.48)	0.25 (0.18 to 0.39)

All values are expressed as the mean ± standard deviation, as well as median and interquartile range (IQR), logarithm of the minimum angle of resolution visual acuity. There were statistically significant differences in the median CVA values of all 15 colors between the following age groups: 20s and 40s; 20s and 60s; 30s and 60s (Kruskal-Wallis test, *p*<0.01, 0.01, and 0.001, respectively).

### Comparison of the variances of the CVA values between the 15 colors

**Fig 5:**
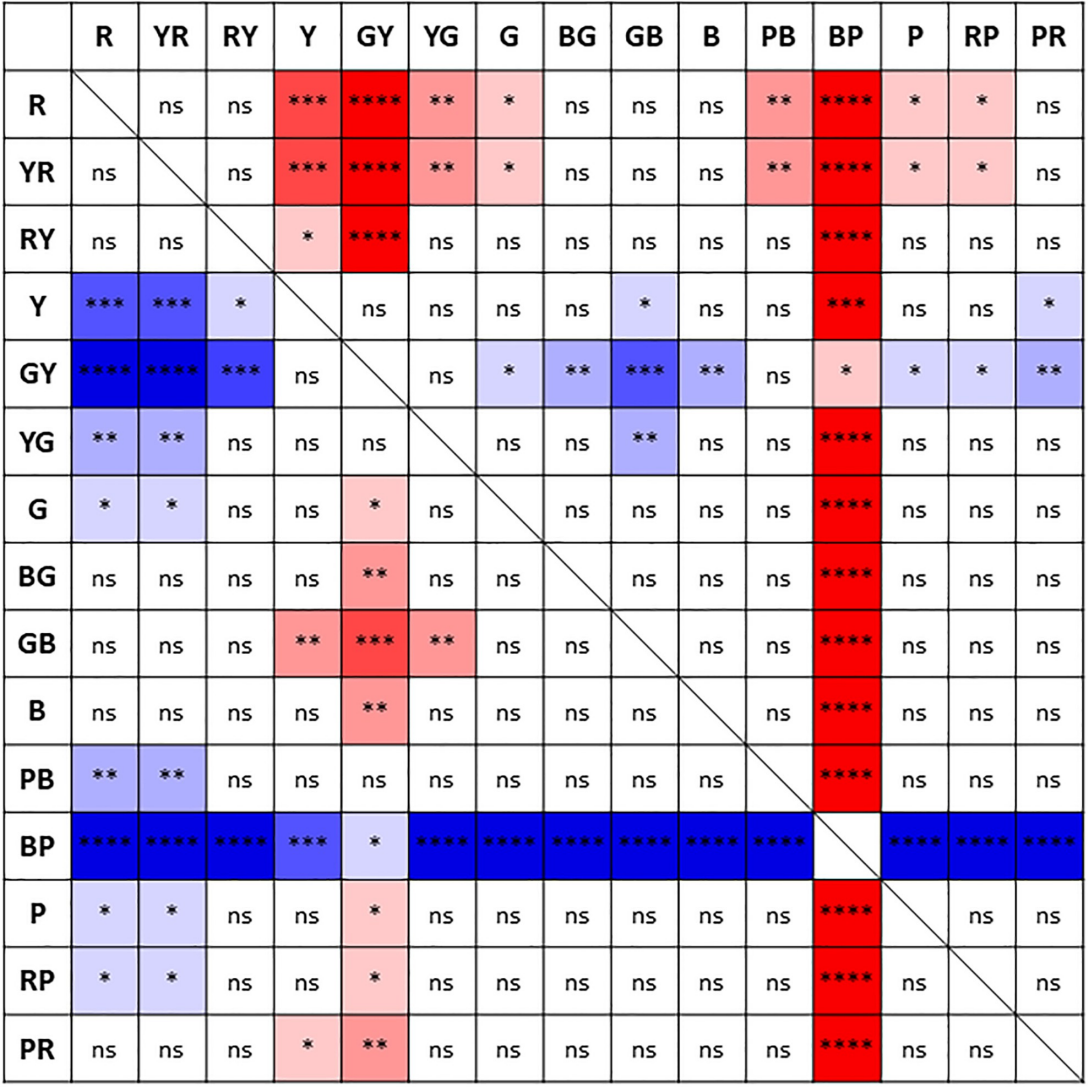
Comparison of the variances of the color visual acuity (CVA) values between the 15 colors in all participants. The *p*-values of the F-test for comparison between variances in CVA values for the 15 colors in all participants are summarized (ns: no significant difference, *p<0.05, **p<0.01, ***p<0.001, ****p<0.0001). The cells are shown in warmer colors (red shades) when the colors arranged vertically have significantly smaller variances in CVA values than those of the colors arranged horizontally, and the cells are shown in cooler colors (blue shades) when those variances are significantly larger. R: red, YR: yellow-red, RY: red-yellow, Y: yellow, GY: green-yellow, YG: yellow-green, G: green, BG: blue-green, GB: green-blue, B: blue, PB: purple-blue, BP: blue-purple, P: purple, RP: red-purple, PR: purple-red.

The colors with the smallest to largest SD values of CVA in all participants were as follows: R, YR, GB, RY, PR, BG, B, G, RP, P, PB, YG, Y, GY, and BP. Fig 5 summarizes the comparison of variances of the CVA values between the 15 colors in all participants. The smallest variance in CVA value was obtained for R, which was significantly smaller than for Y, GY, YG, G, PB, BP, P, and RP. The highest variance in CVA value was obtained for BP, which was significantly larger than for all other colors.

### Comparison of CVA values between age groups

The age groups from best to worst mean CVA value (of all 15 colors) were as follows: 20–29 (mean ± SD, 0.303 ± 0.113), 30–39 (0.324 ± 0.096), 10–19 (0.333 ± 0.022), 50–59 (0.335 ± 0.078), 40–49 (0.339 ± 0.096), and 60–69 (0.379 ± 0.125) years. There were statistically significant differences in median CVA values of all 15 colors between the following age groups: 20s and 40s; 20s and 60s; 30s and 60s (Kruskal-Wallis test, all *p*<0.01). S12–S17 Figs illustrate the 15 median (interquartile range) CVA values for each age group.

## Discussion

In this study, although BCVA measured by using the conventional black-and-white VA test was not correlated with age, the CVA values of nine colors (R, YR, RY, G, BG, GB, P, RP, and PR) were negatively correlated with age. On the other hand, the CVA values of six colors (Y, GY, YG, B, PB, and BP) were not correlated with age. Among the nine colors for which the CVA values were negatively correlated with age, four (R, G, BG, and PR) were located close to the protanopic (P) or deuteranopic (D) confusion lines, which relate to the M/L-cones (Fig 1A). On the other hand, among the six colors for which the CVA values were not correlated with age, GY and BP were located close to the tritanopic (T) confusion line, which relates to the S-cones (Fig 1A). Additionally, the CVA values of R, G, BG, and PR were higher and less varied than those of GY and BP (Figs 4 and 5). These results suggest that the CVA values of colors located on the P or D confusion lines were higher, less varied, and more susceptible to aging than the CVA values of colors located on the T confusion line. Knoblauch et al. [16] evaluated the chromatic sensitivity along the P, D, and T confusion lines in normal eyes by using an equiluminant color change of a set of bars embedded in a larger field of spatio-temporal achromatic noise. In their study, the chromatic sensitivity on the P, D, and T confusion lines increased from infancy to around 20 years of age, and then decreased with age. Paramei et al. [17] evaluated the results of the Cambridge Colour Test (CCT), which uses colored Landolt ring targets similar to those of the CVA test, on normal eyes of participants in their teens to 80s. The color discrimination on the P, D, and T confusion lines was best in participants in their thirties and thereafter decreased with age, and the chromatic sensitivity on the T confusion line decreased statistically significantly after the age of 60 years. In these reports, in addition to the sensitivity to colors on the P and D confusion lines, the sensitivity of colors to the T confusion line also decreased with age. However, in our current study, the CVA values for colors on the T confusion line were not influenced by age. The reason for this discrepancy may be related to the difference in the range of foveal cone function evaluated with each examination. The visual angle of the stimulus used in the report of Knoblauch et al., was 7°[16], and the visual angles of the Landolt ring and the gap size in the CCT were 5° and 1°, respectively [17]. On the other hand, the visual angles of the Landolt ring and the gap size in our study were 1.3° and 0.26° (equivalent to a logMAR VA of 1.2), respectively, even at the maximum presented size. Thus, it is possible that the CVA test evaluates the cone function of a more central part of the fovea compared with previously reported tests. Curcio et al. [18] examined the cone distribution and morphology of human cone photoreceptors by using a human donor eye. They reported that S-cones were missing in a zone of about 0.35° around the foveal center and that the S-cones were irregularly distributed around the edges of that zone. This may be associated with the current results of lower CVA values and larger variances of CVA values for colors on the T confusion line, which relate to the S-cones, than those for colors on the P and D confusion lines, which relate to the M/L-cones. Therefore, it is possible that the CVA values for colors on the T confusion line would not be affected by aging. In addition, in the chromatic sensitivity tests used in those previous studies, the threshold values were measured by changing the saturation (chroma) without changing the visual-stimulus size [16, 17]. On the other hand, CVA was measured at a certain fixed saturation value (chroma 6) in this study, and the threshold value was measured by changing the stimulus size. Hence, it is also possible that the differences in the examination methods affected the results.

There are various possible reasons for the decline in color vision with age. Pupil size, crystalline lens coloration, and macular pigment density can influence color vision performance [19–23]. It has been speculated that the major factors of age-related decline in color vision associated with the S-cones are a decrease in short wavelength transmittance due to crystalline lens yellowing, and a decrease in the S-cone response [23–25]. In particular, the yellowing of the crystalline lens exerts a larger effect on the S-cones compared with that on the M/L-cones [26]. In contrast, regarding the mechanism for a specific decline in color vision related to the M/L-cones, several early studies suggested that glaucoma causes an acquired RG-axis color deficiency [6]. In addition, in a study of open-angle glaucoma using a printed CVA chart, among the CVA values for R, BG, GY, and BP, those for BG and R were statistically significantly lower than those in normal eyes [27]. From these results, the age-related decline in the M/L-cones may also be due to the decline in optic nerve function with age, and further studies are needed in this area.

In this study, the 20–29-year age group exhibited the highest mean CVA and the 60–69-year age group exhibited the lowest mean CVA. In a study in which 232 normal eyes (109 men and 123 women) with a BCVA ≥20/20 were evaluated among participants aged 10–80 years by using the F-M 100-hue test, the mean score was the lowest in participants aged 20–29 years [28]. Similarly, in another study, individuals aged 20–50 years had the best F-M 100-hue test scores and those aged 20–55 years had the best Lanthony Desaturated Panel D-15 test scores in 115 normal eyes (63 women and 52 men; aged 5–81 years) with a BCVA of 20/30 or better [29]. In our previous study in which we used SPP-3, one of the pseudoisochromatic plate tests that is used for detection of congenital and acquired color vision deficiency, color vision was evaluated in 23565 normal eyes (participants aged 5–89 years) with a BCVA of 20/20 or better [30]. Therein, we reported that the highest number of correct answers were obtained in participants aged 10–29 years. According to the stimulus size, the color arrangement tests and pseudoisochromatic plate tests performed in those previous reports were used to evaluate the function of the macular area. Yet, although, in the current study, we evaluated the function of the foveal center by means of CVA tests, the best values were obtained in patients in their 20s, which was similar to the previous reports.

There were several limitations in the current study. Although congenital color vision deficiency was excluded by using SPP-3, it is possible that we included participants with congenital color vision deficiency who passed the test. Second, the number of participants was different for each age group, and the number of participants in their teens, fifties, and sixties was relatively small. Also, participants ranged in age from their teens to their sixties, and it is necessary to examine the CVA values in older people for a more detailed evaluation of changes in color perception with age. In the current study, the required number of subjects was determined as 160 according to the statistical analysis in the preliminary study. However, the CVA values of colors on the T confusion line (GY and BP) were lower than those of other CVA values, and their variances were larger. Therefore, it is possible that the sample size was insufficient to statistically analyze the correlation between CVA values for colors on the T confusion line and age.

## Conclusions

In this study, the CVA test was used to measure the chromatic spatial acuity of the foveal center in normal eyes of subjects between 10 to 69 years of age. The CVA was measured at a certain fixed saturation value (chroma 6) in this study, and the threshold value was measured by changing the stimulus size using 15 different color Landolt rings. As a result, younger subjects tended to have higher CVA values than middle-aged and elderly subjects. The CVA values for R, YR, RY, G, BG, GB, P, RP, and PR were susceptible to aging, of which R, PR, RP, G, and BG are located close to the P or D confusion lines, which are related to the M/L-cones. On the other hand, the CVA values for GY and BP, of which are located close to the T confusion line and are related the S-cones, were not affected by aging. The results of the present study differed from those of previous studies, which showed that the color sensitivity of all P, D, and T confusion lines decreased with age after adolescence. The reasons for this discrepancy may be related to the difference in the range of foveal cone function evaluated and the detection methods of the threshold values in the chromatic sensitivity with each examination.

## Supporting information

S1 Video Demonstration of a red and a blue-purple Landolt ring displayed sequentially in the color visual acuity (CVA) testing system. The colored Landolt ring is presented with its gap in one of four directions: up, down, right, or left. If the subject answers correctly, the stimulus size is reduced by one step. If the answer is incorrect or not supplied, the stimulus size is increased by one step. The CVA value is calculated from the minimum Landolt ring size for which the subjects answer correctly more than two times out of a maximum of five times.
(MP4)

S1 Fig The relationship between red-yellow color visual acuity (RY-CVA) values and age for all 162 participants. Among all participants, RY-CVA was negatively correlated with age (Spearman’s correlation coefficient [*r*] = 0.2476, *p* = 0.0015).
(TIF)

S2 Fig The relationship between yellow color visual acuity (Y-CVA) values and age for all 162 participants. Among all participants, Y-CVA was not correlated with age (Spearman’s correlation coefficient [*r*] = 0.0940, *p* = 0.2341).
(TIF)

S3 Fig The relationship between yellow-green color visual acuity (YG-CVA) and age for all 162 participants. Among all participants, YG-CVA was not correlated with age (Spearman’s correlation coefficient [*r*] = 0.1255, *p* = 0.1116).
(TIF)

S4 Fig The relationship between green color visual acuity (G-CVA) and age for all 162 participants. Among all participants, G-CVA was not correlated with age (Spearman’s correlation coefficient [*r*] = 0.1884, *p* = 0.0164).
(TIF)

S5 Fig The relationship between blue-green color visual acuity (BG-CVA) and age for all 162 participants. Among all participants, BG-CVA was not correlated with age (Spearman’s correlation coefficient [*r*] = 0.1641, *p* = 0.0369).
(TIF)

S6 Fig The relationship between green-blue color visual acuity (GB-CVA) and age for all 162 participants. Among all participants, GB-CVA was not correlated with age (Spearman’s correlation coefficient [*r*] = 0.2193, *p* = 0.0051).
(TIF)

S7 Fig The relationship between blue color visual acuity (B-CVA) and age for all 162 participants. Among all participants, B-CVA was not correlated with age (Spearman’s correlation coefficient [*r*] = 0.0984, *p* = 0.2130).
(TIF)

S8 Fig The relationship between purple-blue color visual acuity (PB-CVA) and age for all 162 participants. Among all participants, PB-CVA was not correlated with age (Spearman’s correlation coefficient [*r*] = 0.0632, *p* = 0.4244).
(TIF)

S9 Fig The relationship between purple color visual acuity (P-CVA) and age for all 162 participants. Among all participants, P-CVA was not correlated with age (Spearman’s correlation coefficient [*r*] = 0.2117, *p* = 0.0068).
(TIF)

S10 Fig The relationship between red-purple color visual acuity (RP-CVA) and age for all 162 participants. Among all participants, RP-CVA was correlated with age (Spearman’s correlation coefficient [*r*] = 0.2055, *p* = 0.0087).
(TIF)

S11 Fig The relationship between purple-red color visual acuity (PR-CVA) and age for all 162 participants. Among all participants, PR-CVA was correlated with age (Spearman’s correlation coefficient [*r*] = 0.2432, *p* = 0.0018).
(TIF)

S12 Fig The 15 color visual acuities in the teenager group. The median and interquartile range logarithm of the minimum angle of resolution visual acuity are plotted.
(TIF)

S13 Fig The 15 color visual acuities in the 20s age group. The median and interquartile range logarithm of the minimum angle of resolution visual acuity are plotted.
(TIF)

S14 Fig The 15 color visual acuities in the 30s age group. The median and interquartile range logarithm of the minimum angle of resolution visual acuity are plotted.
(TIF)

S15 Fig The 15 color visual acuities in the 40s age group. The median and interquartile range logarithm of the minimum angle of resolution visual acuity are plotted.
(TIF)

S16 Fig The 15 color visual acuities in the 50s age group. The median and interquartile range logarithm of the minimum angle of resolution visual acuity are plotted.
(TIF)

S17 Fig The 15 color visual acuities in the 60s age group. The median and interquartile range logarithm of the minimum angle of resolution visual acuity are plotted.
(TIF)
