# Supplementary figures and images for: Age-related changes of color visual acuity in normal eyes
Source: PLoS One. 2021 Nov 29;16(11):e0260525. doi: 10.1371/journal.pone.0260525 (PMC8629265; doi:10.1371/journal.pone.0260525)

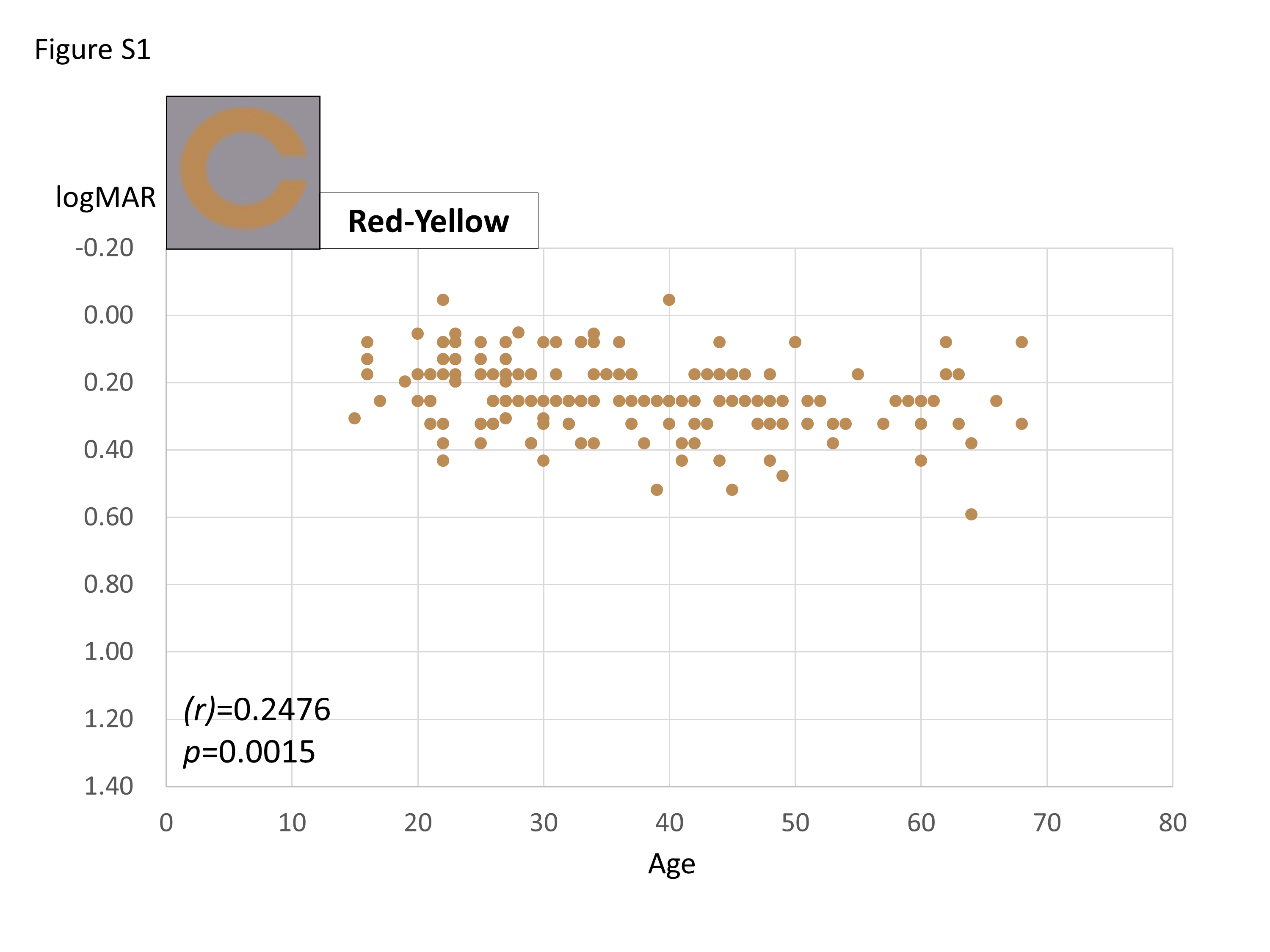

Supplement: S1 Fig — Among all participants, RY-CVA was negatively correlated with age (Spearman’s correlation coefficient [r] = 0.2476, p = 0.0015). (TIF) [file pone.0260525.s002.TIF]

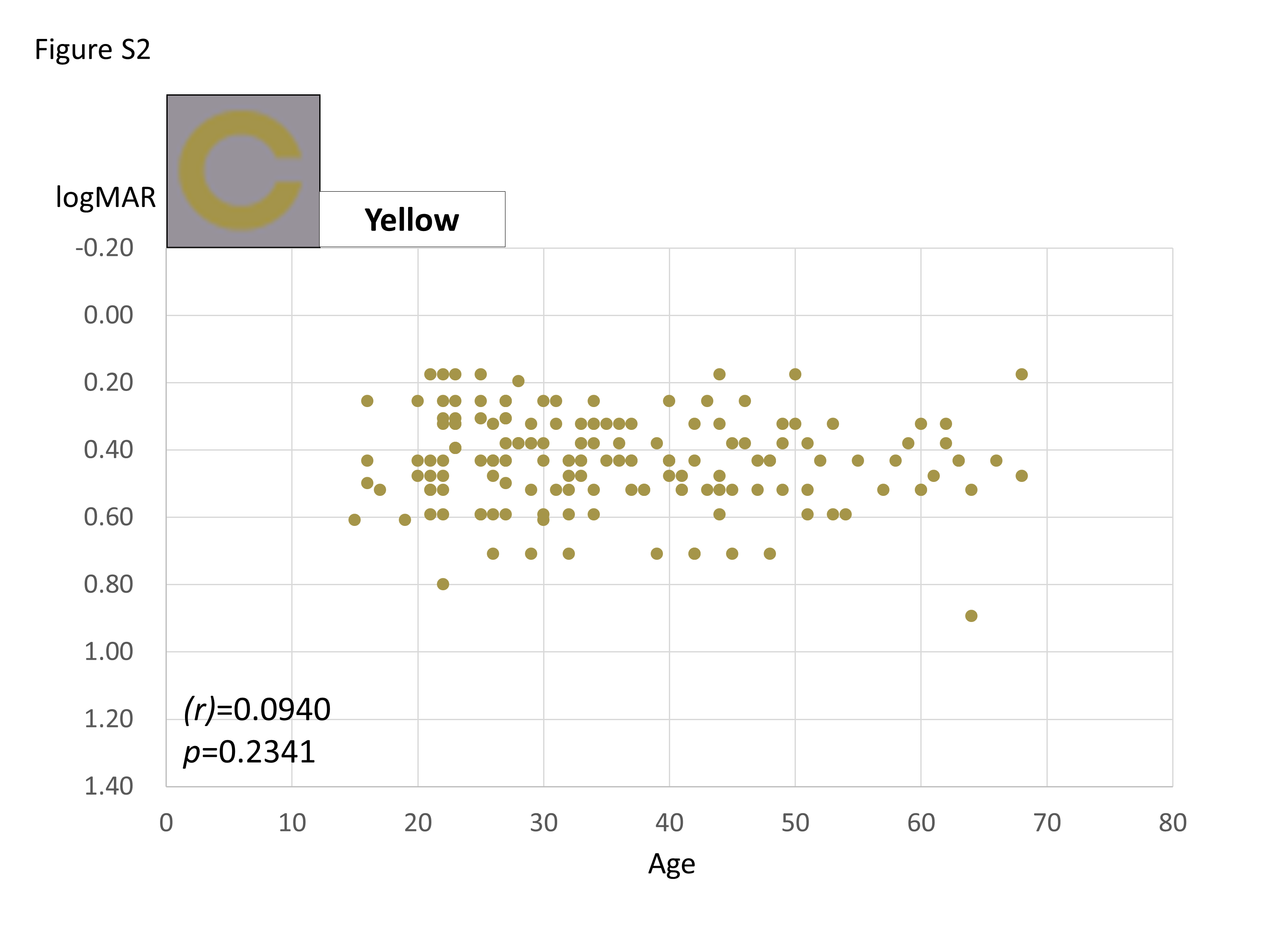

Supplement: S2 Fig — Among all participants, Y-CVA was not correlated with age (Spearman’s correlation coefficient [r] = 0.0940, p = 0.2341). (TIF) [file pone.0260525.s003.TIF]

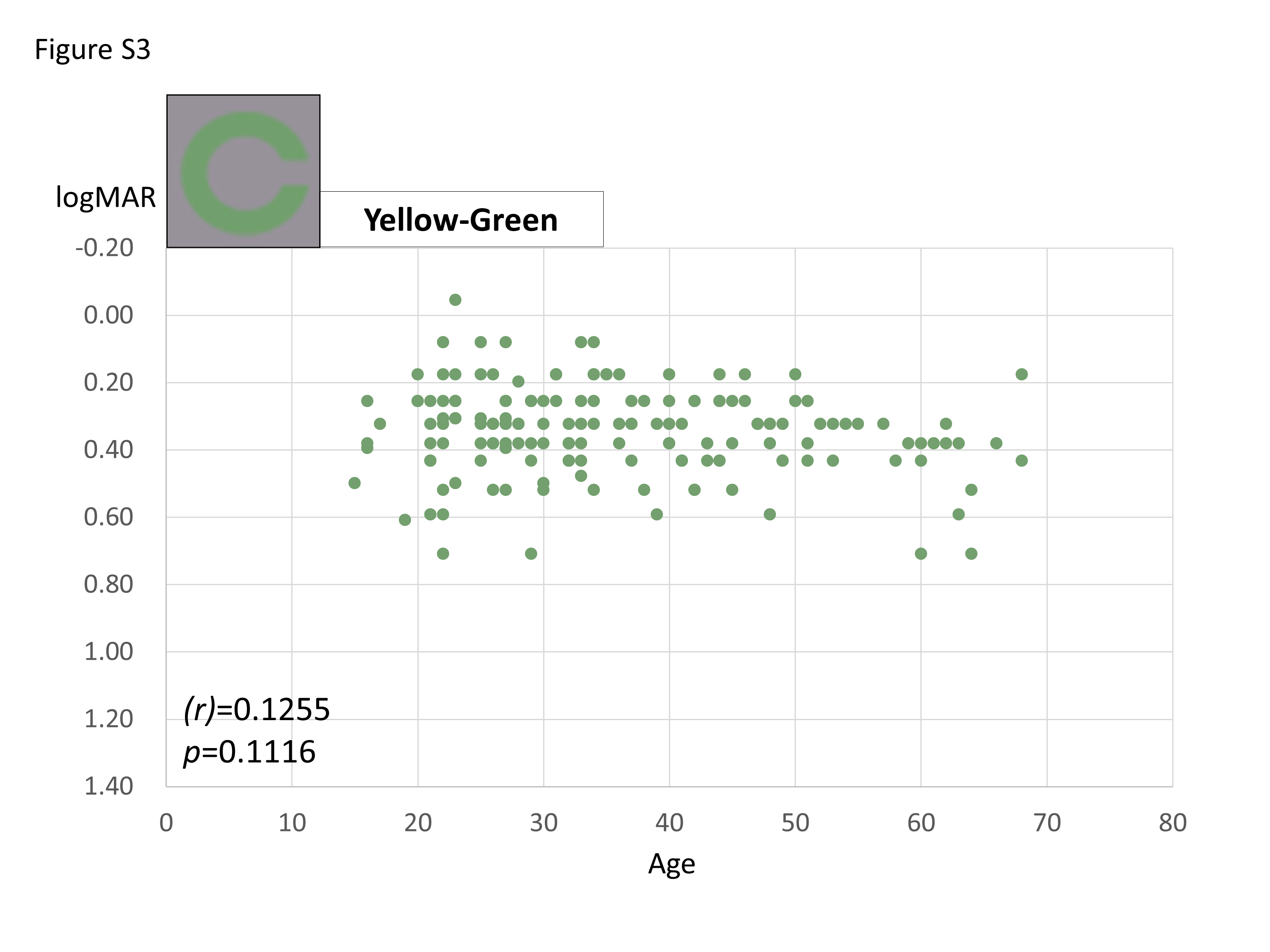

Supplement: S3 Fig — Among all participants, YG-CVA was not correlated with age (Spearman’s correlation coefficient [r] = 0.1255, p = 0.1116). (TIF) [file pone.0260525.s004.TIF]

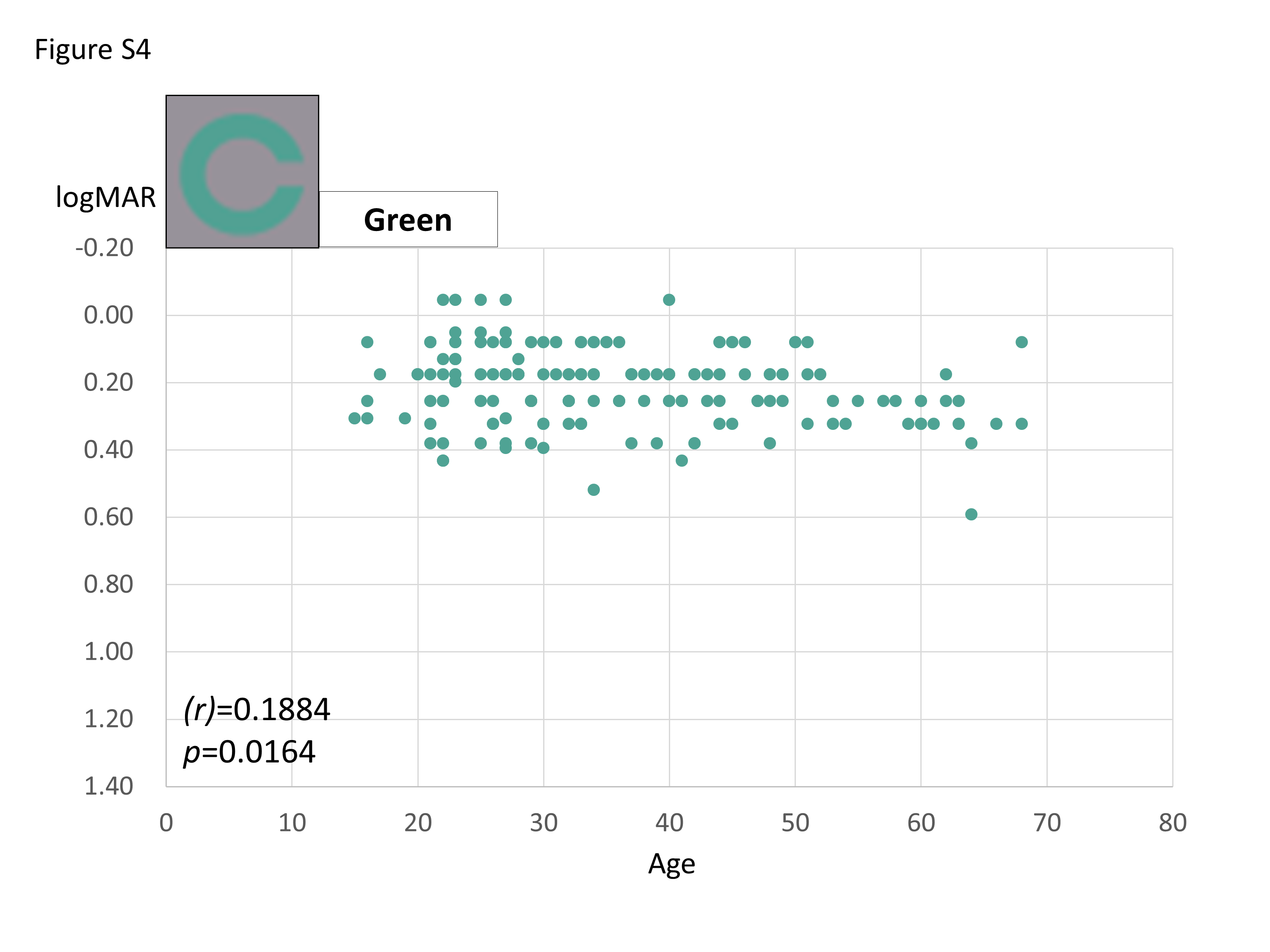

Supplement: S4 Fig — Among all participants, G-CVA was not correlated with age (Spearman’s correlation coefficient [r] = 0.1884, p = 0.0164). (TIF) [file pone.0260525.s005.TIF]

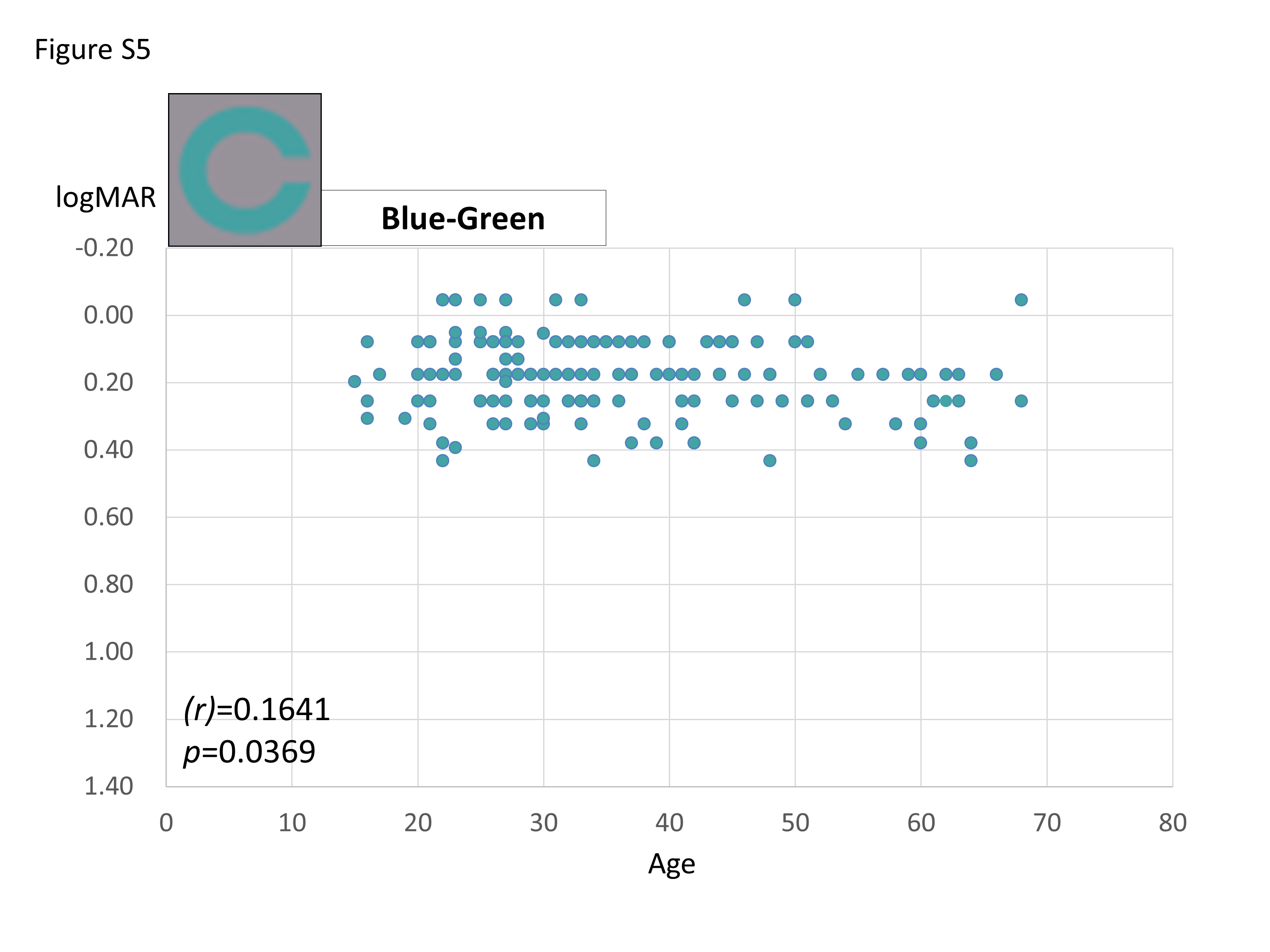

Supplement: S5 Fig — Among all participants, BG-CVA was not correlated with age (Spearman’s correlation coefficient [r] = 0.1641, p = 0.0369). (TIF) [file pone.0260525.s006.TIF]

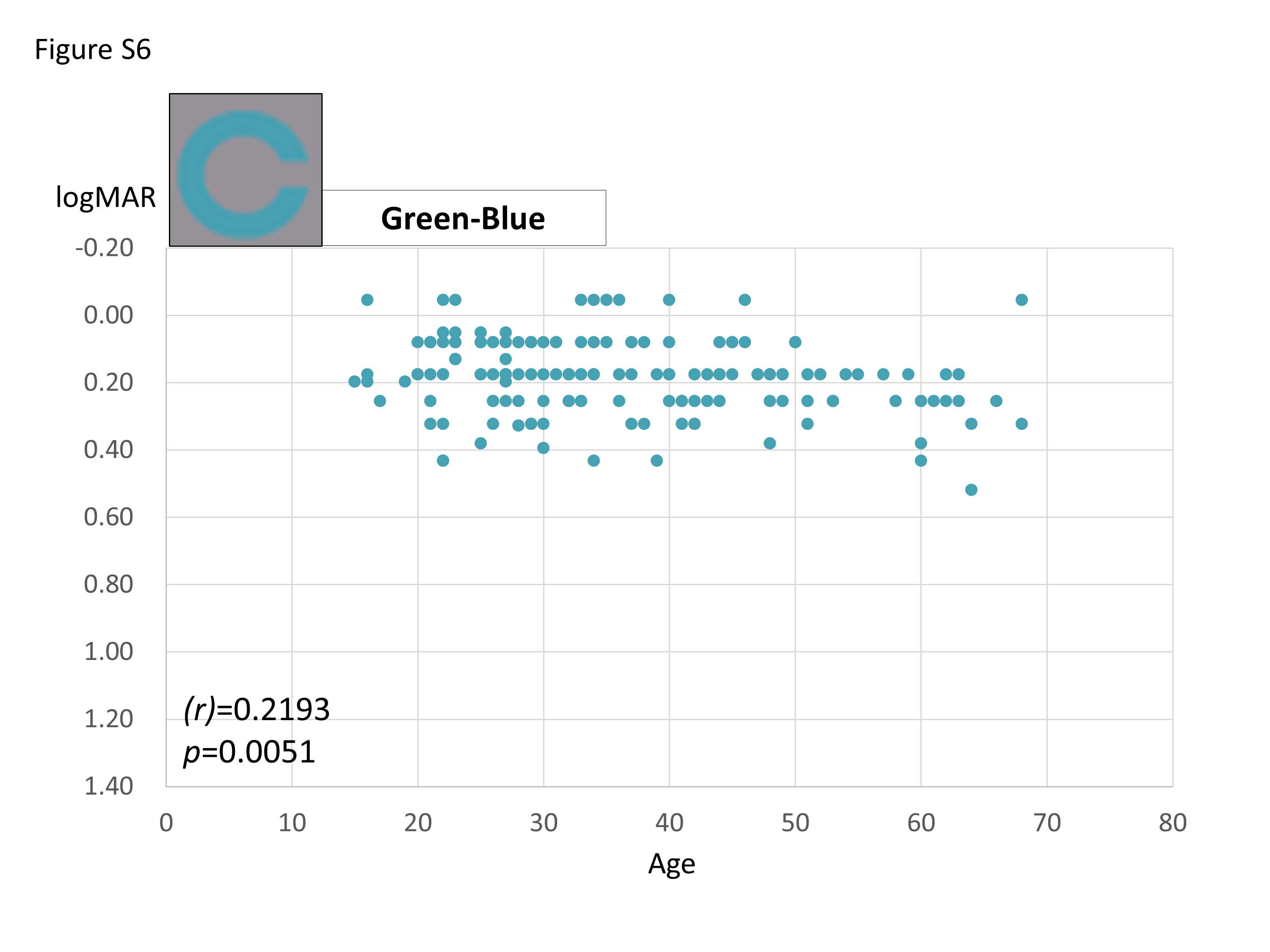

Supplement: S6 Fig — Among all participants, GB-CVA was not correlated with age (Spearman’s correlation coefficient [r] = 0.2193, p = 0.0051). (TIF) [file pone.0260525.s007.TIF]

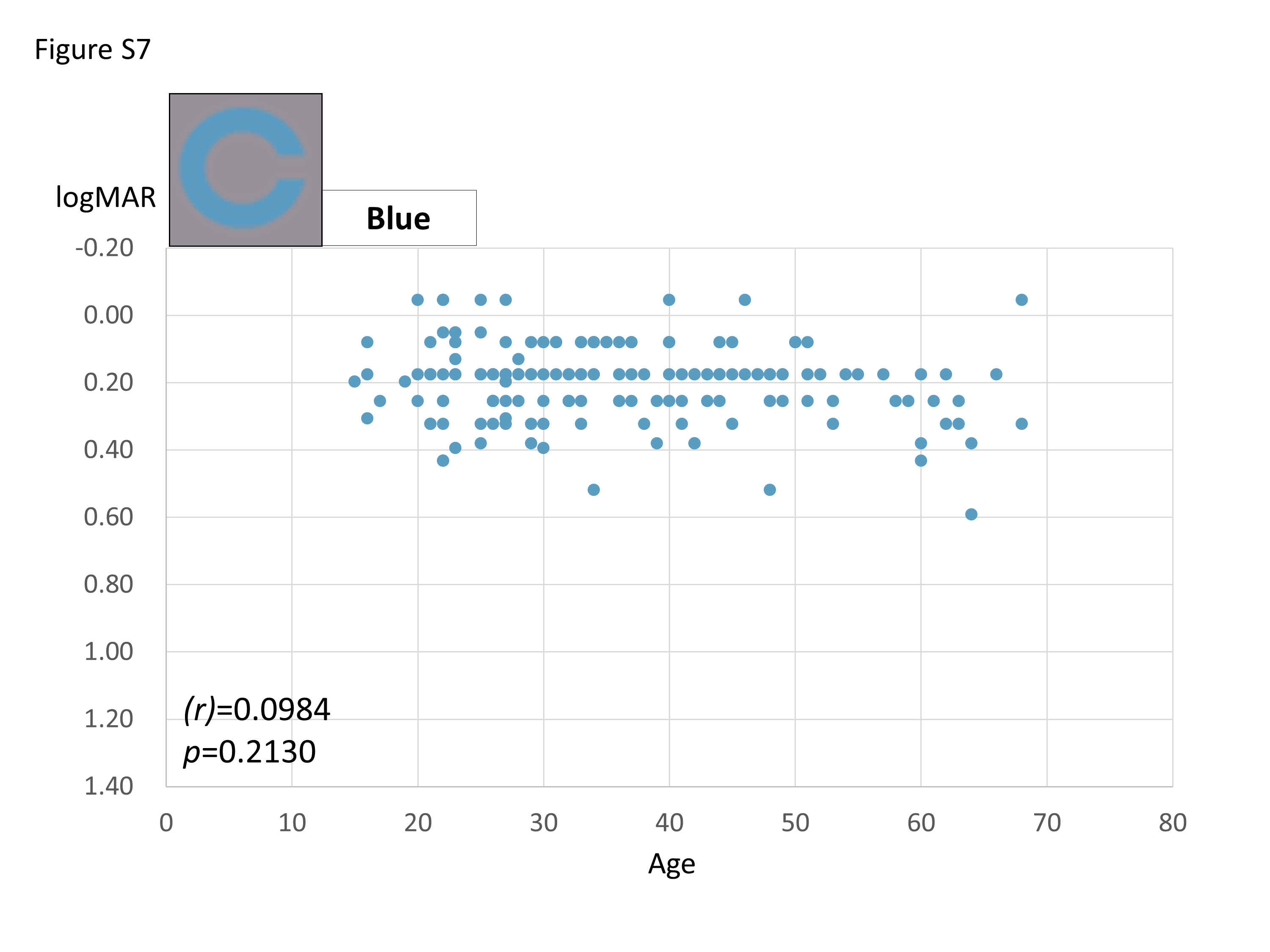

Supplement: S7 Fig — Among all participants, B-CVA was not correlated with age (Spearman’s correlation coefficient [r] = 0.0984, p = 0.2130). (TIF) [file pone.0260525.s008.TIF]

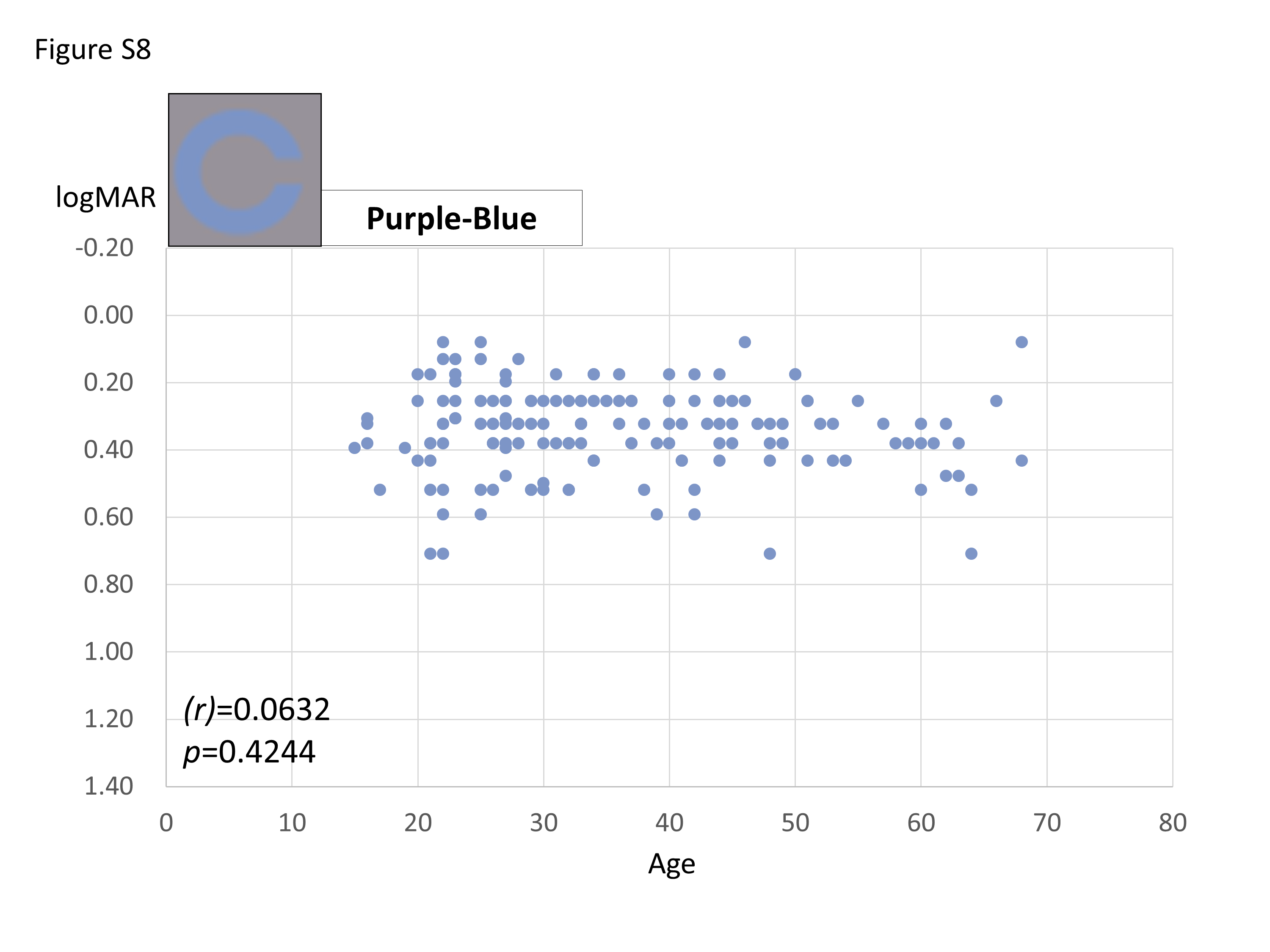

Supplement: S8 Fig — Among all participants, PB-CVA was not correlated with age (Spearman’s correlation coefficient [r] = 0.0632, p = 0.4244). (TIF) [file pone.0260525.s009.TIF]

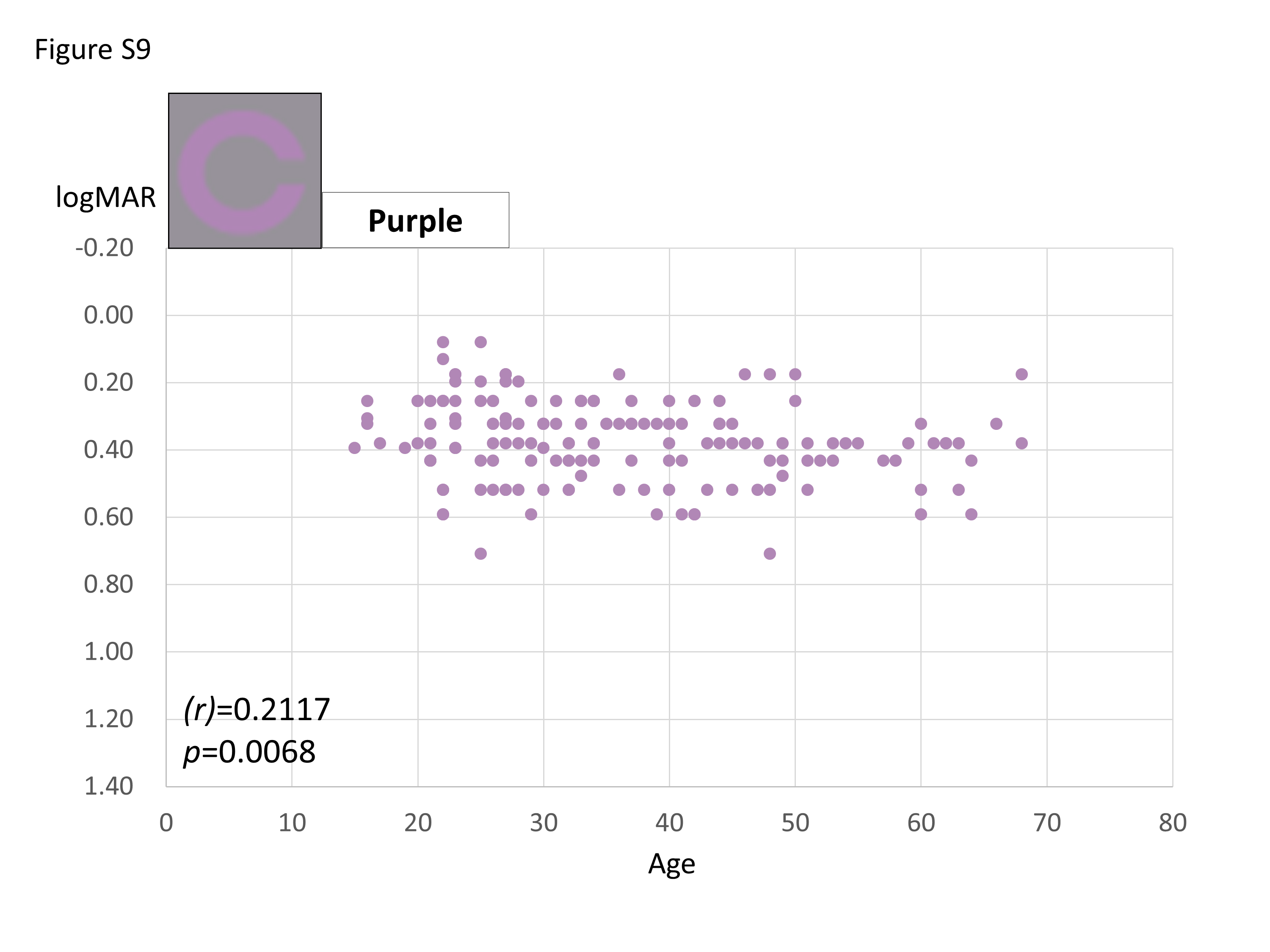

Supplement: S9 Fig — Among all participants, P-CVA was not correlated with age (Spearman’s correlation coefficient [r] = 0.2117, p = 0.0068). (TIF) [file pone.0260525.s010.TIF]

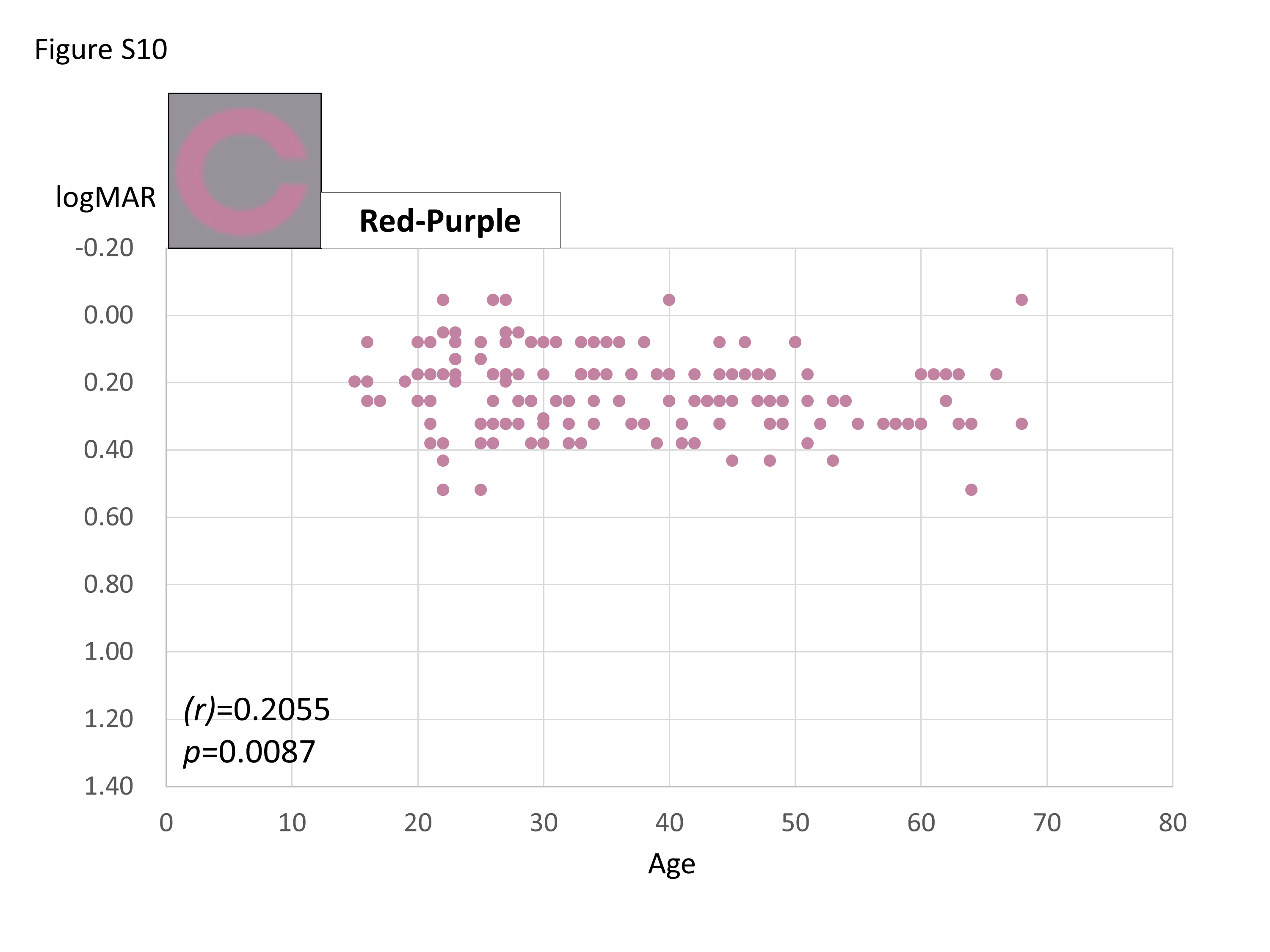

Supplement: S10 Fig — Among all participants, RP-CVA was correlated with age (Spearman’s correlation coefficient [r] = 0.2055, p = 0.0087). (TIF) [file pone.0260525.s011.TIF]

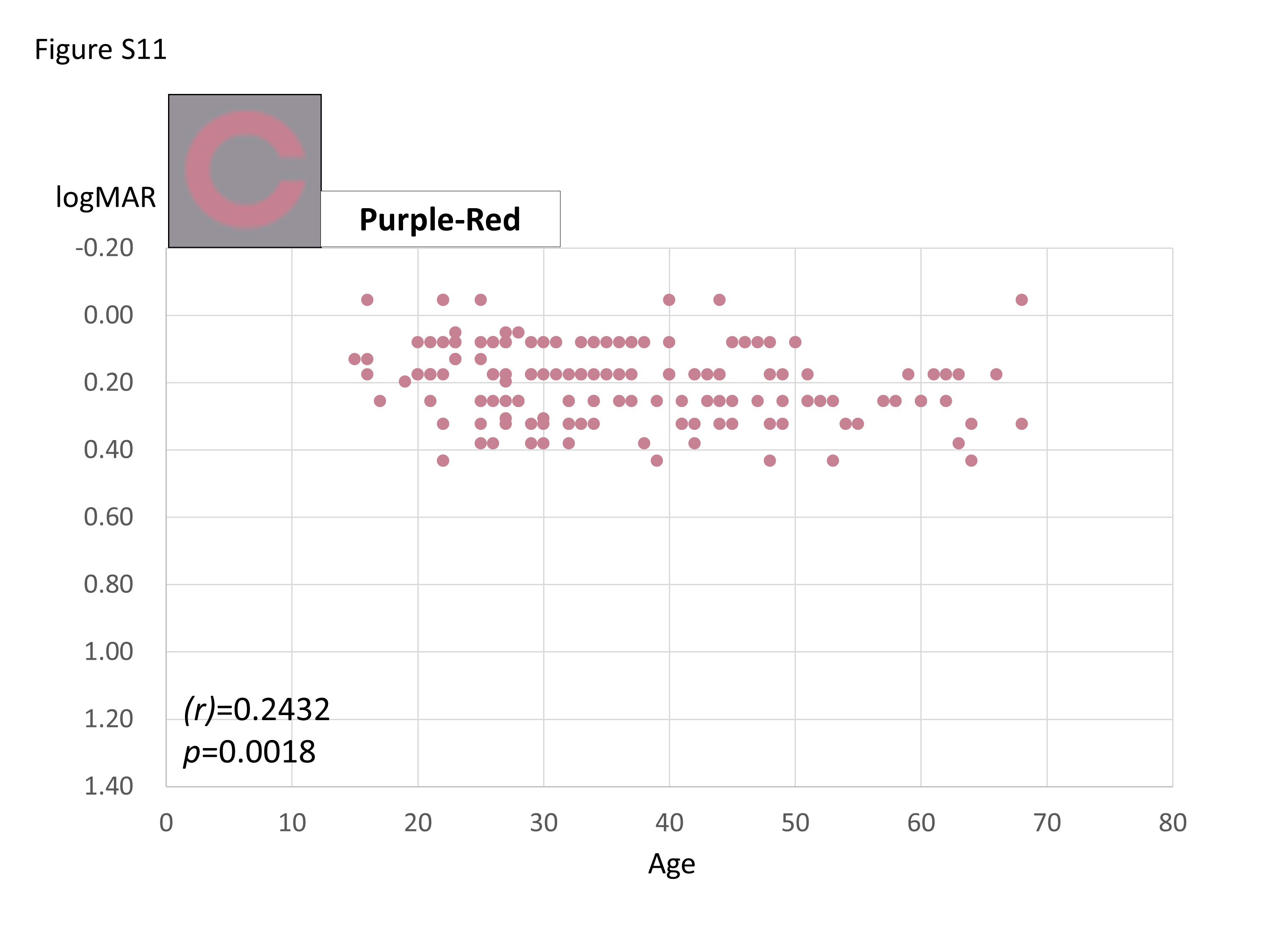

Supplement: S11 Fig — Among all participants, PR-CVA was correlated with age (Spearman’s correlation coefficient [r] = 0.2432, p = 0.0018). (TIF) [file pone.0260525.s012.TIF]

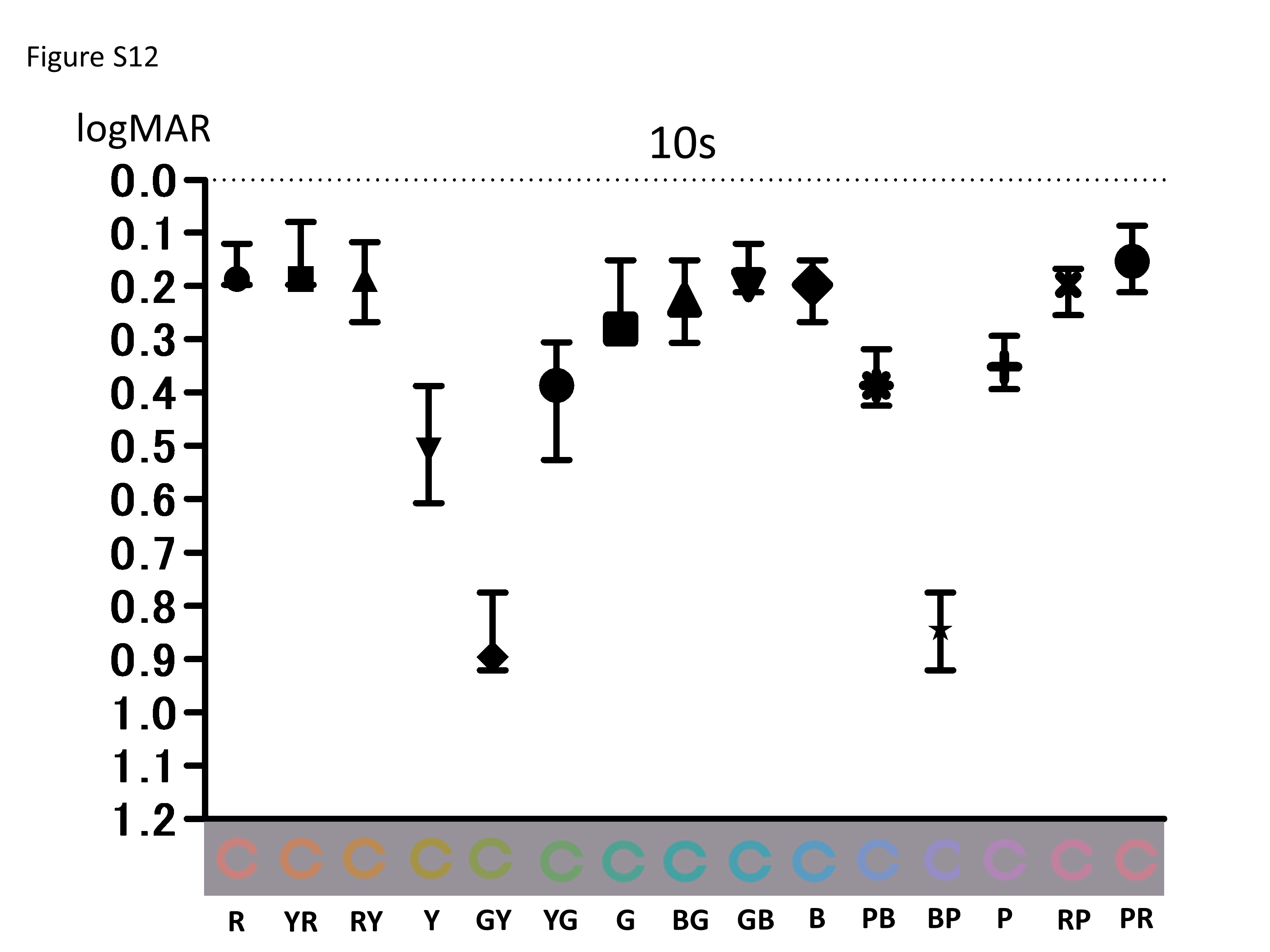

Supplement: S12 Fig — The median and interquartile range logarithm of the minimum angle of resolution visual acuity are plotted. (TIF) [file pone.0260525.s013.TIF]

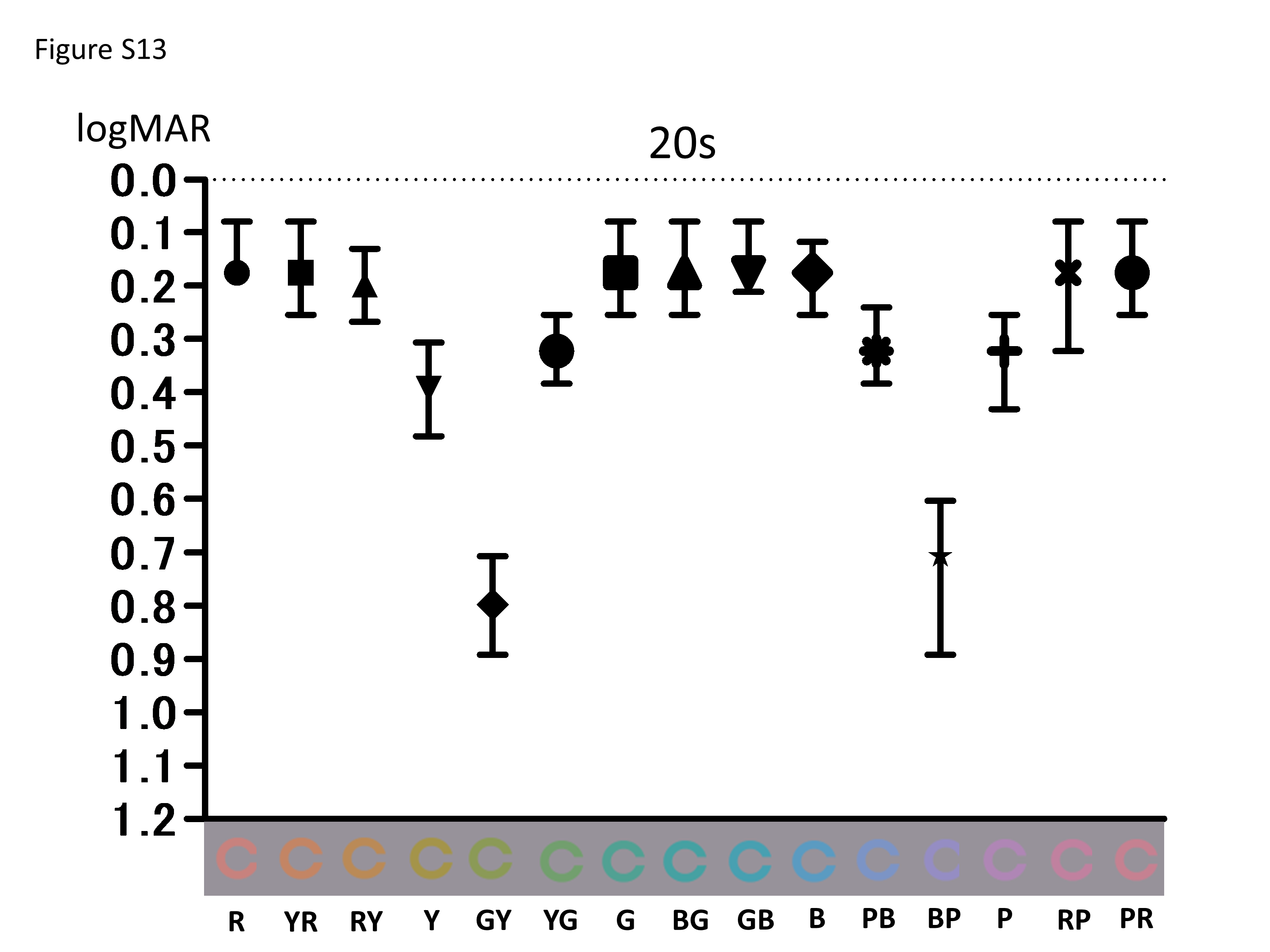

Supplement: S13 Fig — The median and interquartile range logarithm of the minimum angle of resolution visual acuity are plotted. (TIF) [file pone.0260525.s014.TIF]

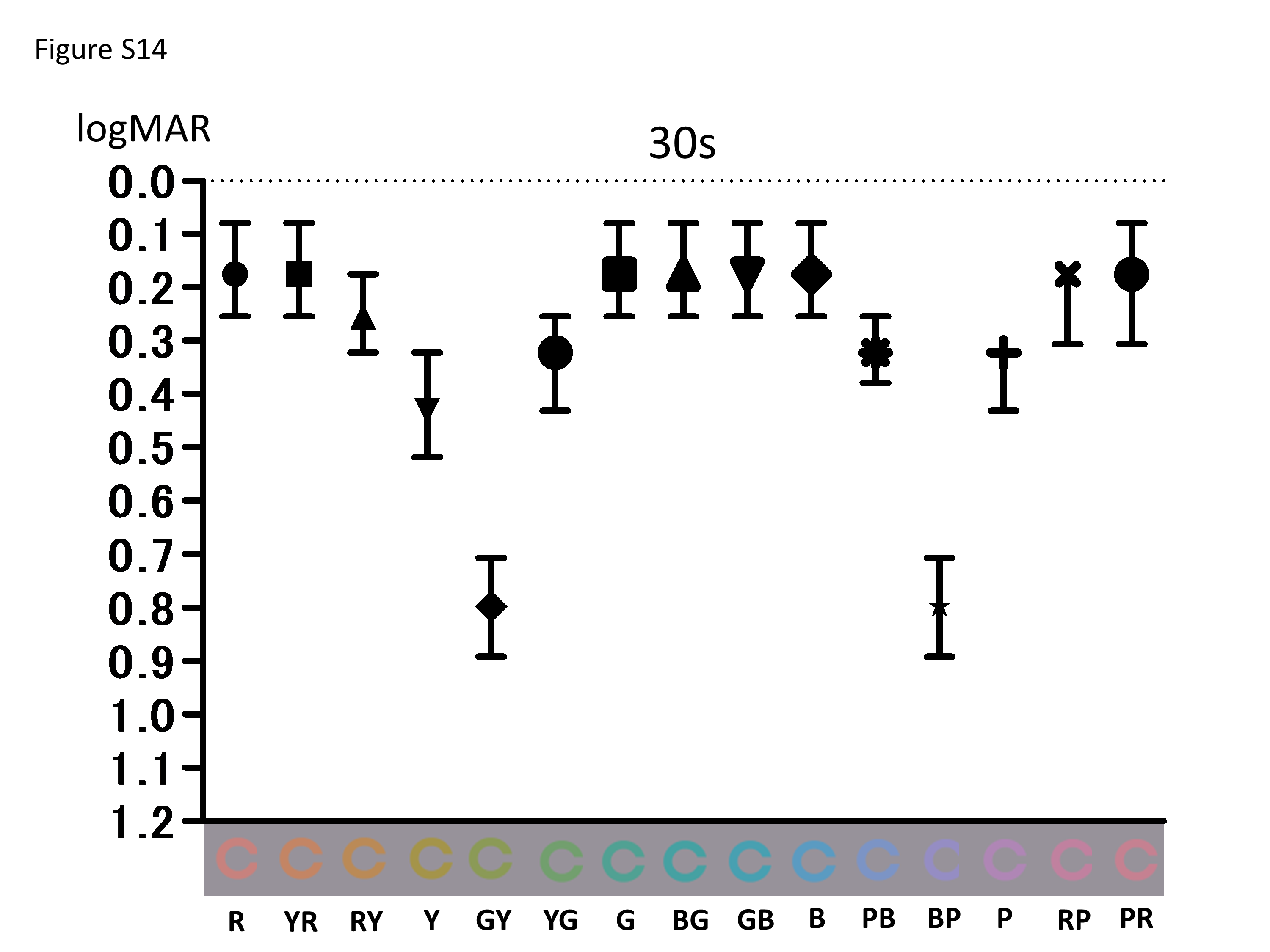

Supplement: S14 Fig — The median and interquartile range logarithm of the minimum angle of resolution visual acuity are plotted. (TIF) [file pone.0260525.s015.TIF]

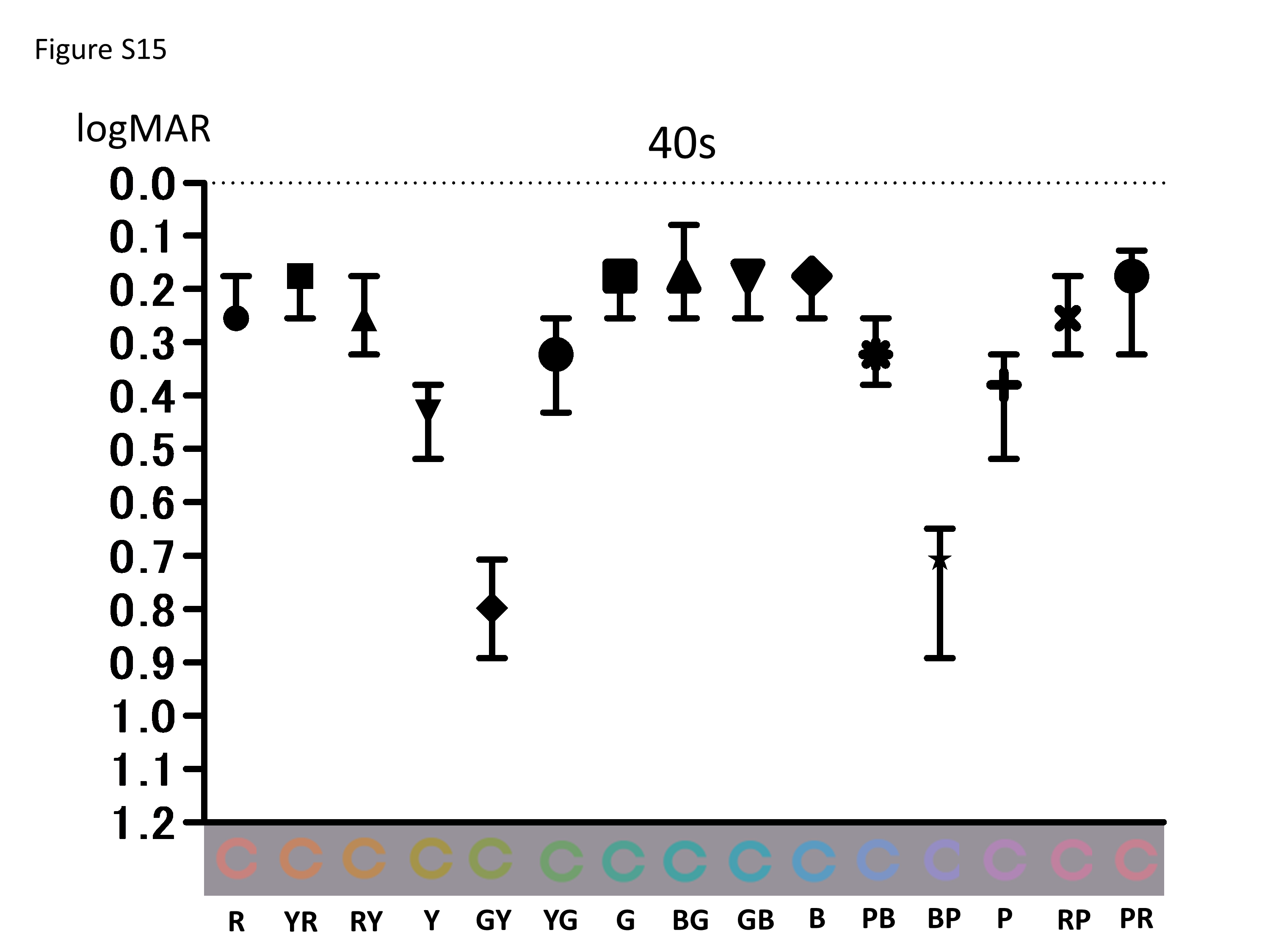

Supplement: S15 Fig — The median and interquartile range logarithm of the minimum angle of resolution visual acuity are plotted. (TIF) [file pone.0260525.s016.TIF]

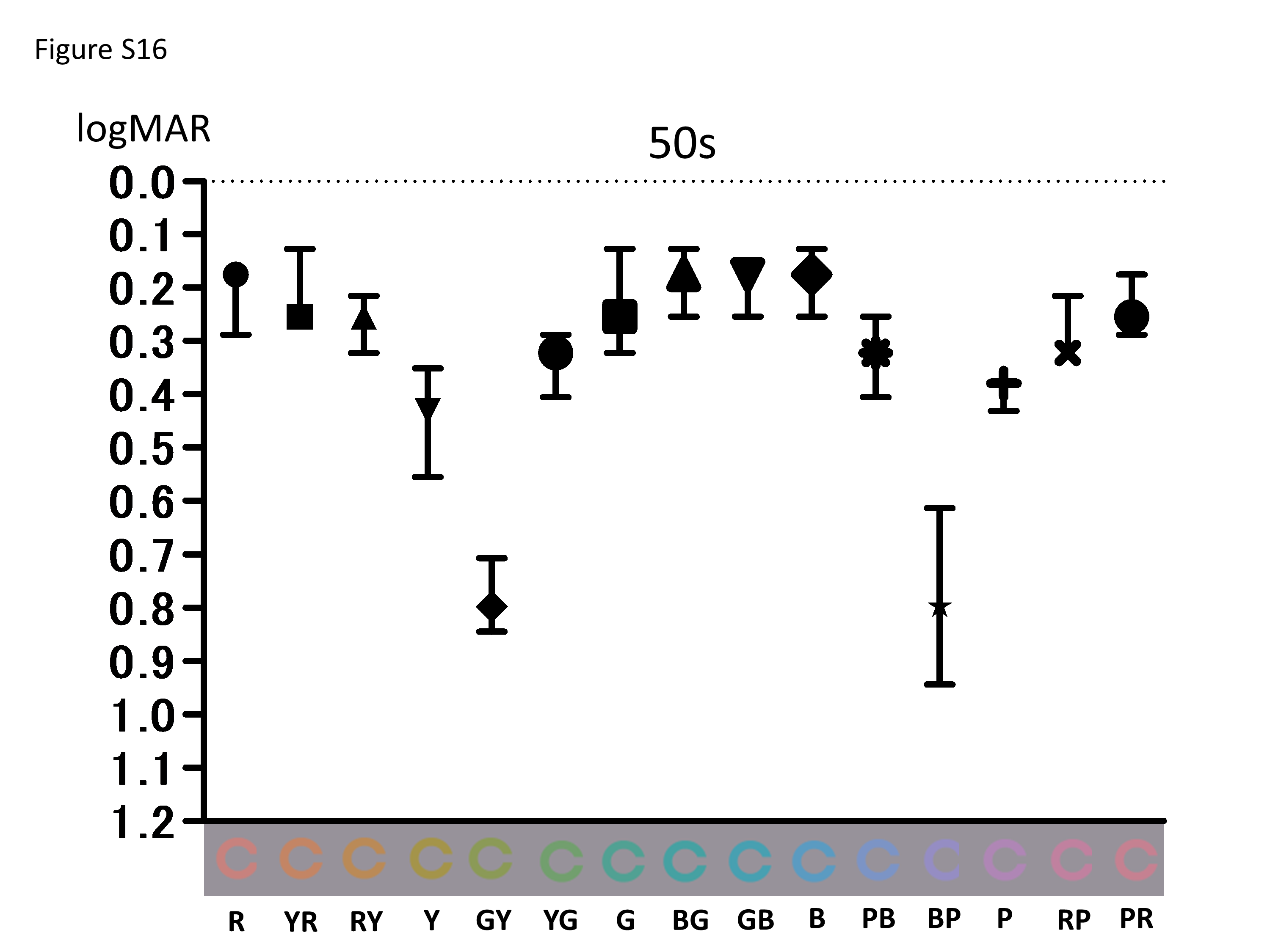

Supplement: S16 Fig — The median and interquartile range logarithm of the minimum angle of resolution visual acuity are plotted. (TIF) [file pone.0260525.s017.TIF]

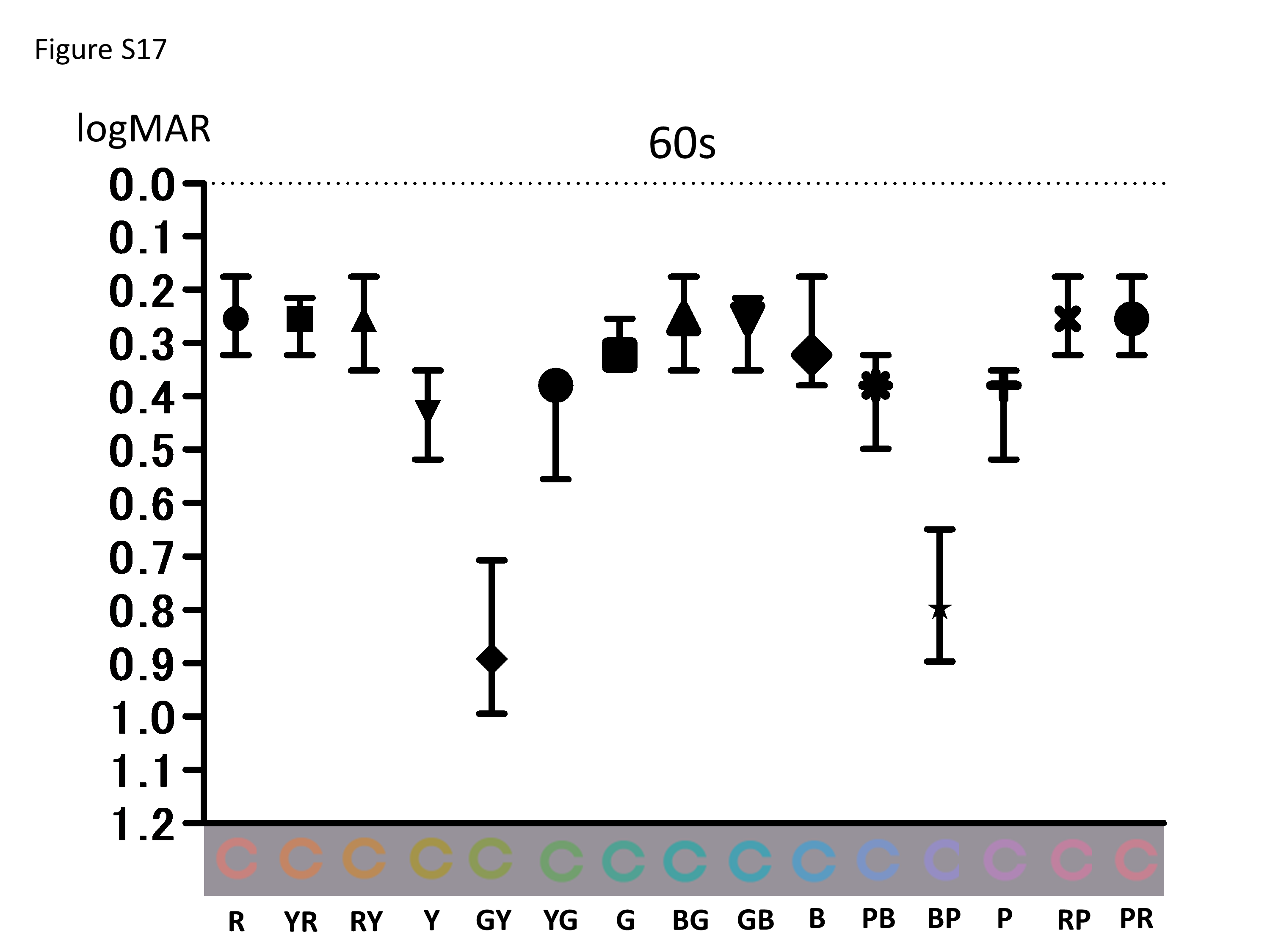

Supplement: S17 Fig — The median and interquartile range logarithm of the minimum angle of resolution visual acuity are plotted. (TIF) [file pone.0260525.s018.TIF]
